# European household spending and socio-economic impacts on food behavior during the first wave of COVID-19

**DOI:** 10.3389/fnut.2022.869091

**Published:** 2022-08-03

**Authors:** Hristo Hristov, Jeremy Millard, Igor Pravst, Meike Janssen

**Affiliations:** ^1^Nutrition Institute, Nutrition and Public Health Research Group, Ljubljana, Slovenia; ^2^Third Millennium Governance, Ry, Denmark; ^3^International Center, Danish Technological Institute, Taastrup, Denmark; ^4^Biotechnical Faculty, University of Ljubljana, Ljubljana, Slovenia; ^5^VIST–Faculty of Applied Sciences, Ljubljana, Slovenia; ^6^Consumer and Behavioral Insights Group, Copenhagen Business School, Frederiksberg, Denmark

**Keywords:** food consumption, food purchasing, COVID-19, financial status, household composition, behavioral change

## Abstract

This paper provides a European-level analysis using a large-scale survey of 13 countries to examine the power of relevant economic and socio-demographic characteristics to account for changes in food consumption and purchasing behavior during COVID-19. This was done by focusing on a two-level analysis of subject-related predictors highlighted in many existing country-level studies to test the generality of their significance. The Level 1 predictors relate to the individual households participating in the survey consisting of household composition, education, and location, as well as three types of perceived COVID-19 risks of infection, severity, and anxiety. Level 2 relates to the national level, and especially to the financial situation measured by the mean national Actual Individual Consumption (AIC) per capita in PPP, of the countries, in which the households reside. In terms of changes in food consumption, results show that household composition, education, and the household’s perceived risk of both being infected by COVID-19 and being severely infected are significant predictors, although there are some differences between the two levels. Some possible explanations are as follows: putting food into one’s body in the context of the pandemic is related to a household’s financial situation, its composition, especially the presence or absence of children and older people, and its educational attainment, and through all these aforementioned to the perception of COVID-19 infection and its severity risks. Changes in food purchasing react significantly to the same predictors, but additionally, to all other predictors at both household and AIC levels. The household’s location and perceived COVID-19 anxiety risks are thus also significant. Food purchasing depends much more on factors operating both at the individual household level and the AIC level together; for example, households’ access to food is affected by both national and local lockdown restrictions that vary according to the location of the household.

## Introduction

### Introduction and structure of the paper

The first wave of the COVID-19 pandemic that started in March 2020 had widespread and severe impacts in terms of lockdowns, closures, and restrictions on both economic and social life across the whole of Europe. Even so, there were important differences in detail between countries and regions in terms of when and how these measures were applied by both national and regional authorities (1, 2). These policy and regulatory differences were reflected in variations in the access to, and consumption of, food by households and their behavioral responses. This was further complicated by the continent’s varied food systems, food cultures, political systems, economic conditions, socio-economic and cultural characteristics, agricultural practices, and climate zones. Hence, many important differences are observed between countries, as reflected in the “Literature review” section.

However, also as apparent from the literature review, there are many similarities between countries when viewed on a larger European scale, two of the most important of which are in focus in this paper drawing on a consumer behavior survey of 13 countries: Czechia, Denmark, France, Germany, Greece, Hungary, Ireland, Israel, Italy, the Netherlands, Serbia, Slovenia, and the United Kingdom. First, an assessment of the general financial situation of the population using a monetary measure of consumption based on national Actual Individual Consumption (AIC) data before the pandemic as a predictor of food security or vulnerability during the pandemic. Second, the household composition and, particularly, the presence, or otherwise, of children. The significance of these two proposed predictors is tested in this paper in relation both to food purchasing and food consumption, while not ignoring other potential predictors, which likely contribute to the food behavioral changes seen.

The paper is structured into four main sections. First, this Introduction lays out the overall context and purpose of the paper, provides a literature review relevant to this purpose, and states the paper’s main aims. The section on “Materials and methods” describes the sample used, how data collection takes place and the limitations of this, explains how the data are analyzed, and articulates the conceptual framework underpinning how these materials and methods are deployed. The “Results” section looks, first, at the descriptive statistics of four country groups based on their AIC data in relation to COVID-19 restrictions, risk perceptions, and six national cultural dimensions. Second, it undertakes a modeling analysis of changes in food consumption and purchasing in relation to the AIC groups and three categories of household composition. Third, the “Results” section also examines the model estimated changes in food consumption and purchasing in relation to the AIC groups and the three categories of household composition. Finally, the “Discussion” section draws out and discusses some overall conclusions about the importance of different types of predictors and possible explanations for the results seen.

### Literature review

A large amount of literature has already examined the impact of COVID-19 on food systems and consumer behavior. In a survey of households in Denmark, Germany, and Slovenia, Janssen et al. (3) found that between 15 and 42% changed their food consumption patterns during the first wave of COVID-19 and that this was related to the closure of physical places to eat outside the home, reduced shopping frequency, individuals’ perceived risk of COVID-19, income losses due to the pandemic, and socio-demographic factors including household composition. In a German study, Profeta et al. (4) showed that COVID-19 had a significant impact on consumers’ eating habits that generally led to negative health consequences, especially amongst economically vulnerable groups, including households that lost income during the pandemic, and those with children. The purchase of ready meals and canned food increased, including the consumption of alcohol and confectionery, at the same time as there was a decrease in the purchase of high-quality and more expensive food like vegetables and fruits. Similar patterns are seen in the state of Vermont in the United States where the utilization of food banks was more common among food-insecure households and households with children. Many food-insecure respondents were also significantly more likely to report consuming fewer fruit and vegetables during the pandemic (5). Similarly, Millard et al. (2) showed that households that lost income during the pandemic were much more likely to grow their food and to obtain free food in food banks. Capodistrias et al. (6) outlined how in 2020, compared to 2019, European food banks redistributed a significantly higher amount of food despite numerous social restrictions and other challenges associated with the pandemic.

A study in Denmark found that a substantial proportion of respondents (≥ 28%) reported eating more, snacking more, exercising less, and gaining weight during the lockdown (7). Results could be linked to the amount of time spent at home (e.g., a higher cooking frequency) and a higher degree of emotional eating during the lockdown (e.g., higher consumption of pastries and alcohol). Two studies in Italy showed, first, that during the first phase of COVID-19 people increased their interest in and appreciation of food, as well as of environmental, human, and animal welfare issues (8). The second Italian study showed, that although the amount of food consumed during the pandemic increased, food waste declined as people moved to more non-perishable food and away from fresh food products (9).

A meta-analysis of COVID-19-induced changes in food habits in Italy, France, Spain, Portugal, and Poland indicated the generally negative effect of quarantine on eating habits and physical activity with an increase in food consumption and reductions in physical activity, as well as consequential weight gain (10). An analysis of consumer spending data largely focused on Australian and American markets, charted the potential increase of negative psychological effects during the pandemic, like panic buying, herd mentality, and changing discretionary spending (11). In a survey of 54 countries from January to April 2020, Taylor (12) found that pandemics often give rise to the panic buying of groceries and other supplies, especially when people are told to go into self-isolation. This can spread *via* social media showing images and videos of people panicking and emptying shelves in shops, leading to a snowball effect where anxiety and fear of scarcity create real but short-term scarcity. In an Italian survey, Di Renzo et al. (13) showed that physical distancing and self-isolation strongly impact the lives of the citizens by affecting their eating habits and everyday behavior. The two major impacts include staying at home (leading to digital education, smart working, limited outdoor activity, and in-gym physical activity) and stockpiling food due to the restrictions on grocery shopping. There are also generational effects, as demonstrated by Eger et al. (14) in Czechia during the second wave of COVID-19. Baby Boomers (born between 1946 and 1964 and currently between 58 and 76 years old), Generation X (born 1965-1979/80 and currently 42-57), and Generation Y (born 1981-1994/6 and currently 26-41) each changed their shopping behavior in distinctive ways related to their specific fears. During the crisis, all consumer types tended to focus on their most basic needs, so for the Baby Boomer generation, fears for health played an important role, whereas, for both Generations X and Y, job loss fears were the most important. All three generations had similar fears about their general economic situation.

Valaskova et al. (15) show that the pandemic has affected every aspect of consumer behavior: their expenses, investments, and financial reserves, as well as their financial and social wellbeing. A sample of 425 Slovak respondents was analyzed to reveal the most important factors impacting consumers’ financial situations, as well as effects on the maintenance of new shopping habits established during the pandemic period. The results revealed that consumers’ income, age, and sector of occupation play important roles in the context of new shopping patterns. Similar findings are noted by Jay et al. (16) in the United States, where a strong negative relationship was found between neighborhood income and physical movement. Individuals in high-income neighborhoods increased their days at home substantially more than did the individuals in low-income neighborhoods. Residents of low-income neighborhoods were more likely to work outside the home and have generally faced many more barriers to physical distancing.

Based on a sample of 456 Italian consumers, Russo et al. (17) investigated both the short-term and long-term effects on consumers’ dietary decisions during the first wave of the pandemic emergency. They looked at changes in food purchases, respondents’ mood during the lockdown, conspiracist beliefs, exposure to the virus, and planned food purchasing behavior after the lockdown. Two opposite approaches to changes in food purchasing decisions were identified: an impulsive approach and a reflective approach, with the former demonstrating a higher probability of changing food purchases but a lower probability to keep these changes over the longer term. Results suggest that COVID-19 psychological pressure was associated with an impulsive approach to buying food. Consequently, food-purchasing behavior is expected to revert to pre-COVID-19 habits when the emergency is over. In contrast, Millard et al. (2) analyzing data from 12 European countries showed that, during the pandemic, income-loss-households are more likely than other households to state that some of the positive changes they have made and were, perhaps, forced to make, during COVID-19 are more likely to continue post-pandemic. These include significant increases in shopping with local producers and in more local shops, growing their food, and using a wider range of food dishes and recipes. However, it is unclear whether the reason for this expectation by income-loss households is that they can see the benefits of such changes which in some, but by no means all, cases are already practiced by no-income-loss households, or because they expect their relatively precarious situation will persist regardless of the state of the pandemic.

It has long been noted that boredom and stress can lead to over-eating, especially “comfort food” with a high sugar content that increases serotonin intake leading to a positive effect on mood (18). It is now clear that a further acceleration of these behaviors has been driven by COVID-19 alongside a reduction in fresh fruit and vegetable consumption and, as noted above, these pandemic-induced trends are seen especially in more financially vulnerable households given their more tenuous links to the labor market and greater likelihood of infection, and thus higher potential stress levels (3, 19). Indeed, Millard et al. (2) revealed the high importance of whether households lost income during the pandemic and that this is a good surrogate for individual household income. Despite the fact that all categories of the household during COVID-19 increased both the amount of food eaten and the amount of money spent on food, income-loss households were more likely to do this despite their financial fragility even before the pandemic, which then made their situation worse. Income-loss households nearly always experienced food behavior changes arising from COVID-19 much more than no-income-loss households, probably because their financial and social situations are more precarious, so they are more sensitive to external shocks and are likely to react more strongly under stress. The precariousness of income-loss-households is also related to the fact that they are overrepresented in regions with the lowest PPP/inhabitant, have a lower mean age, and are more likely to be families with children, which together imply both lower earning potential and that finances need to be stretched further.

### Aims of this paper

The above literature review starkly demonstrates the often dramatic changes in food-related behaviors during COVID-19 and that economically and socially vulnerable consumers seem to be affected by the pandemic much more than others. Indeed, there is very strong evidence that households already experiencing some financial vulnerability were pushed to even greater precariousness during the pandemic, thereby, further exacerbating food vulnerability, and related inequalities. The literature review also underlines the importance of household composition in influencing COVID-19-induced food behavior changes.

However, given that much of the existing literature focuses mainly on single countries or small groups of countries, this paper’s relatively large-scale survey of 13 countries aims to analyze relevant economic and socio-demographic characteristics at the European level by focusing on the two main predictors of households’ financial situation and household composition. Thus, the 13 countries are grouped according to their mean AIC per capita in PPP, as detailed in Table 1. AIC is potentially a relevant perspective on household financial resilience, or lack of such, as it relates directly to the size of their disposable income, as well as influencing the propensity for households to save (20). According to Eurostat (21), food in EU households in 2019 “represents 13% of total consumption expenditure and ranks as the third-largest category of household expenditure after “housing, water, electricity, gas, and other fuels, which accounted for 23.5% of household expenditure, and “transport” (13.1%).” As noted in the literature review, there is also strong evidence that expenditure on food increased during the pandemic. This conclusion is backed by the latest Eurostat data showing that since 2019, expenditure on food increased by 3.2%, communications by 2.4%, and household consumption of energy and water by 0.3%, while all other expenditures decreased, including eating out by –37.8% (22). Most people were stuck at home during lockdowns, so had more time to devote to food and were able to divert some expenditure from transport and entertainment to food, although the frequency of food purchasing decreased due to shopping restrictions.

**TABLE 1:** Description of the sample and population-weighted adjustments.

Country sample	Sampling method	Sample data *N* (%)	Weighted data *N* (%)^a^	AIC per head & PPPs^b^	Allocation to AIC group^c^
Denmark	Quota	1,281 (16.1)	131 (1.6)	34,601	Very high
Germany	Quota	1,020 (12.8)	1,870 (23.4)	36,509	
Netherlands	Convenience	122 (1.5)	389 (4.9)	34,103	
United Kingdom	Convenience	314 (3.9)	1,526 (19.1)	33,866	High
Ireland	Convenience	595 (7.4)	111 (1.4)	28,435	
France	Quota	644 (8.0)	1,489 (18.6)	29,545	
Italy	Convenience	538 (6.7)	1,340 (16.7)	25,935	Low
Israel	Quota	641 (7.7)	197 (2.5)	25,935	
Czechia	Quota and convenience	805 (10.2)	241 (3.0)	25,377	
Slovenia	Quota	683 (8.5)	47 (0.6)	24,608	Very low
Hungary	Convenience	720 (9.0)	218 (2.7)	20,075	
Greece	Convenience	539 (6.7)	252 (3.1)	23,129	
Serbia	Convenience	107 (1.3)	197 (2.5)	15,132	
Total		8,009 (100)	8,009 (100)		

**AIC group^c^**	**Sample data *N* (%)**		**Weighted data *N* (%)^a^**		**Mean (*SD*) AIC per head and PPPs^b^**	

Very low	2049 (25.6)		715 (8.9)		20,736 (4186)	
Low	1984 (24.8)		1,778 (22.2)		25,749 (322)	
High	1553 (19.5)		3,126 (39.0)		30,615 (2869)	
Very high	2423 (30.1)		2,381 (29.9)		35,071 (1270)	

*^a^*Weighted according to each country’s 2020 population: https://data.oecd.org/pop/population.htm. *^b^*AIC is Actual Individual Consumption per head at current prices ($) and purchasing power parity (PPP), 2019: https://www.oecd-ilibrary.org/economics/actual-individual-consumption-price-indices_26ff7815-en (26). *^c^*Quartile segmentation based on country Actual Individual Consumption per capita and PPP ($), 2019.

The aim of the paper is thus to examine the extent to which the variance across the two main food-related behaviors of consumption and purchasing within the whole sample of 13 countries can be explained at two levels: Level 1 of individual survey households, and Level 2 of AIC (a monetary measure of consumption). Various combinations within and between these two levels are examined. The paper thereby aims to fill an important gap in the literature by extending our understanding of how a sudden shock impacts these behaviors.

## Materials and methods

### Sample description and data collection

The evidence base consists of data from a common online questionnaire containing 34 questions that were accessible *via* a dedicated website^1^ and are now available as part of the Supplementary Material. It was designed to capture the changes in respondents’ behavior in relation to food purchasing, preparation, and consumption, as well as experiences of COVID-19-related illness, regulations, and closures. Ancillary information was also collected on household socio-economic characteristics, including households’ income changes from before to during the pandemic. The questionnaire was translated into national languages by local researchers from the 13 countries, providing a good representation of Europe’s varied food systems, food cultures, political systems, economic conditions, socio-demographic characteristics, agricultural practices, and climate zones.

The sampling of respondents combined two methods. First, representative quota samples of respondents based on gender, age, education, and regional distribution (data collection by market research agencies). Second, convenience sampling was deployed, by which respondents were contacted largely *via* social media, although local researchers in these countries attempted to reach out to all main population groups in all parts of the country. We recognize the potential limitations of this dual strategy made necessary because our network of researchers from many countries needed to be established rapidly as the first wave struck, so not all of them were able to quickly ensure enough funding for representative sampling and data collection by market research agencies. In some countries, such agencies were hired but funding was restricted so the quota sampling and data collection were accompanied by some convenience sampling of respondents to boost the sample. However, to minimize any bias we have weighted each country’s sample based on their 2020 population, as indicated in Table 1. In addition, this research study is based on relatively large sample sizes where local researchers endeavored to include as many different population cohorts as possible even when convenience sampling was implemented. Moreover, the questionnaire was entirely consistent across all countries, translated into local languages by local experts, and the analysis does not take place at the individual country level.

The questionnaire responses that were considered invalid, and thus excluded, were those where respondents took less than 5 min to answer or where they had responded incorrectly to attention-check questions in different parts of the questionnaire. These procedures resulted in responses from at least 100 households in each country yielding 8,009 responses in total (see Table 1 for an overview). Data were collected during the first wave from March to July 2020 and then merged into a large dataset of respondents from all 13 countries. Table 1 describes the sampling method, crude, and weighted data per country, as well as how countries were clustered into four groups based upon their populations’ AIC as measured by Eurostat-OECD in terms of Purchasing Power Parity (PPP).

To determine changes in food consumption, participants were asked to report how often they consumed 11 types of fresh food, non-fresh food, convenience food and snack food during and before the pandemic. Food purchasing was analyzed based on the four types of fresh fruit and vegetables, fresh meat and meat products (including fish), other fresh products (bread, milk, cheese, etc.), and other non-fresh food (frozen, canned, pre-cooked, drinks, etc.). The food consumption and purchasing frequency questionnaire contained a six-point scale, each of which was proportionately weighted, comprising the following: less than once a fortnight; between once a week and once a fortnight; once a week; 2–3 times a week; 4–6 times a week; and daily. Participants were also asked whether they had experienced certain changes due to COVID-19, including changes in household income and the closure of their physical workplace. Further questions covered the extent to which households had been afflicted with COVID-19, and their own perceived risk of the disease in terms of infection, severity, and anxiety as shown in Table 2, each with a five-point answer scale from very low to very high. Finally, questionnaire respondents provided data on the demographic details of their households and themselves (The full questionnaire is available in the Supplementary Material).

**TABLE 2:** COVID-19-related risk perceptions and impacts per the AIC group: weighted data analysis.

Variable	Level	Very low AIC	Low AIC	High AIC	Very high AIC
		*N* (%)	*N* (%)	*N* (%)	*N* (%)
COVID risk infection	Low	191 (27.0)	697 (39.2)	1,370 (43.8)	1,051 (43.9)
	Medium	288 (40.7)	799 (44.9)	1,245 (39.8)	1,054 (44.1)
	High	229 (32.3)	283 (15.9)	511 (16.3)	286 (12.0)
COVID risk severity	Low	193 (27.2)	589 (33.1)	1,111 (35.5)	984 (41.2)
	Medium	228 (32.1)	578 (32.5)	1,040 (33.3)	909 (38.0)
	High	289 (40.7)	611 (34.4)	976 (31.2)	498 (20.8)
COVID risk anxiety	Low	229 (32.3)	443 (24.9)	1,206 (38.6)	990 (41.4)
	Medium	252 (35.6)	680 (38.2)	1,148 (36.7)	858 (35.9)
	High	228 (32.1)	656 (36.9)	772 (24.7)	544 (22.7)
COVID infection	Yes	55 (7.8)	89 (5.0)	193 (6.2)	87 (3.6)
COVID isolation	Yes	82 (11.5)	118 (6.6)	231 (7.4)	89 (3.7)
COVID hospitalization	Yes	12 (2.1)	7 (0.4)	17 (0.5)	6 (0.3)

Table 3 provides data on the main range of socio-economic and demographic variables of the sample across the four AIC groups.

**TABLE 3:** Description of the AIC groups socio-economic and demographic: weighted data analysis.

Variable	Category	Very low *N* (%)	Low *N* (%)	High *N* (%)	Very high *N* (%)
Total *N* (%)		715 (100)	1,778 (100)	3,126 (100)	2,381 (100)
Household location^†^	Urban	267 (39.2)	770 (46.9)	1,247 (42.6)	1,188 (53.6)
	Intermediate	252 (37.0)	516 (31.5)	1,296 (44.3)	755 (34.1)
	Rural	162 (23.8)	355 (21.6)	383 (13.1)	272 (12.3)
Mean age (*SD*)	Mean age	31.8 (13.6)	44.7 (13.3)	50.0 (15.1)	49.3 (15.7)
Age groups	18–35	303 (68.4)	454 (25.6)	608 (19.5)	530 (22.2)
	36–49	98 (22.1)	648 (36.5)	870 (27.9)	576 (24.1)
	50–65	35 (7.9)	560 (31.6)	1,099 (35.2)	877 (36.8)
	66 and older	7 (1.6)	112 (6.3)	543 (17.4)	403 (16.9)
Gender	Female	363 (65.4)	1,091 (61.6)	2,105 (67.9)	1,347 (56.6)
	Male	192 (34.6)	680 (38.4)	993 (32.1)	1,031 (43.4)
Education	Lower secondary or equivalent	25 (3.5)	2 (0.1)	128 (4.1)	227 (9.5)
	Upper secondary of equivalent	298 (42.0)	503 (32.8)	714 (22.9)	1,244 (52.0)
	University degree or equivalent	386 (54.5)	1,029 (67.1)	2,277 (73.0)	921 (38.5)
Income change	Income-loss	137 (84.6)	576 (38.9)	27 (3.3)	1,466 (75.5)
	No-income-loss	25 (15.4)	904 (61.1)	787 (96.7)	476 (24.5)
Household composition	Household with children 0–19	86 (15.9)	649 (38.0)	947 (30.8)	564 (23.9)
	Single-person household	84 (15.5)	329 (19.2)	745 (24.2)	717 (30.4)
	Households 2 + adults, no children	372 (68.6)	732 (42.8)	1,384 (45.0)	1,078 (45.7)

^†^This regional typology is taken directly from the Eurostat categorizations across the whole of Europe where further details are given: https://ec.europa.eu/eurostat/statistics-explained/index.php?title=Archive:Regional_typologies_overview#Urban-rural_typology_including_remoteness. The last date this document was edited by Eurostat was 3-11-20 and is now marked as archived, but NUTS-3 categorizations remain available on https://circabc.europa.eu/d/d/workspace/SpacesStore/ea154527-d900-431f-b5a8-97fbea6e4b08/regtyp.xls) and can be used to access all Eurostat’s regional data: https://ec.europa.eu/eurostat/web/regions/data/database. (All accessed November 20, 2021).

In Table 3, there is a greater likelihood for households in the two lower AIC groups to reside in rural locations compared to the two higher AIC groups, which tend to be more urban. The lower AIC groups are also more likely to have younger households than the higher AIC groups, and this is especially marked in the Very Low group. The household composition also reflects these two locational and age observations. The lower AIC groups have fewer single-person households than the higher groups, indicating the higher frequency of older persons living alone, especially in urban areas. Furthermore, the two lower AIC groups taken together are more likely to have experienced income loss during the pandemic, which is probably related to the fact that the greater proportions of younger people in these countries tend to be younger couples without children and to be more vulnerable to an economic shock like COVID-19. Related trends from lower to higher AIC are, however, not seen in the education and gender data, probably because these both record the status of the individual respondent rather than the respondent’s total household, which the other variables represent. As in most questionnaires of this type, respondents answering the questionnaire are more likely to be female with a higher than average education. Thus, these two variables in the sample data do not vary in any consistent manner from the Very Low to the Very High AIC groups, so are unlikely to significantly skew the results across the groups.

### Conceptual framework

**FIGURE 1:**
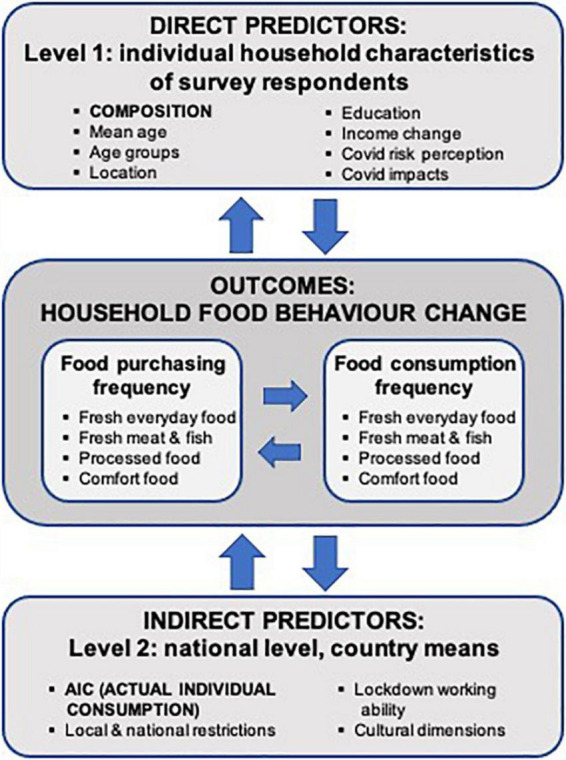
Conceptual framework of both direct and indirect predictors of households’ food change during COVID-19.

Figure 1 sketches the overall conceptual framework indicating how this paper examines food behavior change during COVID-19 in the context of two sets of predictors selected based on the existing literature and the authors’ investigation of the dataset available, as described above. First, there is a set of “direct predictors” at Level 1, so-called as the variables examined consist of data at the individual household level provided by the same respondents reporting their food-related behavioral changes. The main direct predictor of interest is household composition highlighted in capital letters and bold font in Figure 1, while the other direct predictors named are also examined. Second, we examine a set of “indirect predictors” at Level 2, so-called because they are not part of the questionnaire household survey data but are contextual variables collected from reliable sources as explained above. In this case, the main indirect predictor of interest is the national AIC variable, also marked in capital letters and bold font in Figure 1.

Even though AIC is generalized at the national level, the results reported in the “Results” section show it to be the most consistently significant predictor examined. This result was surprising but should not be ignored simply because it is difficult to explain the fact that such a national indicator seems to influence most households in the survey. Reference to Tables 2–5 shows it is strongly related to most socio-economic, demographic, pandemic restrictions, and cultural dimensions, thus, providing an important part of the national setting within which the questionnaire respondents reside and are undoubtedly influenced. We saw from the literature review that income and financial security, in general, were some of the most important predictors of changing food behavior during COVID-19. On this basis, when designing the questionnaire, many partners were keen to ask respondents about their income, as well as their food consumption and purchasing in monetary terms. However, in operational terms, severe constraints arose related to the available time and resources and the level of respondent cooperation required. Thus, it was deemed too challenging to assume that respondents would be able to answer an income question accurately and quickly using the same income and monetary definitions to enable legitimate comparisons across the whole of Europe. Hence, national AIC is used in this paper as a relevant monetary measure of consumption. It is even more powerful than the direct Level 1 predictors, although many of these, including household composition, are also powerful. Another possible reason for the power of national AIC as a predictor is because individual household consumption and income have a strong tendency to be more or less contingent on national economic conditions and policies [e.g., see (21, 22)], especially when we examine large samples of households together, as in this paper. One aim of the paper has been to test this assumption, and the results below do show that there is much credence in doing so.

### Data analysis

Statistical analyses and data management were performed using IBM SPSS Statistics for Windows, version 27 (IBM Corp., Armonk, N.Y., United States). The main predictors (independent variables) and the main outcomes (dependent variables) examined are listed in Figure 1 (see below for further explanations and necessary definitions). Most of the independent variables were direct measures from the questionnaire or were modified by reducing the number of levels to one feasible for analysis, while AIC was created based on the quartile segmentation of each country’s AIC and PPPs per head at current prices ($) (see Table 1). All socio-demographic and household-related responses are reported as counts and frequencies, while lockdown working ability data (see Table 4) and cultural dimension data (see Table 5) are with means and standard deviations (*SD*). The change in food consumption and purchasing were calculated separately for each observed food category as the difference between frequency measured on a six-point scale during and before COVID-19. The determined change for different food consumption and purchasing types was further used in the within-subject analysis under different between-subject conditions. To determine the individual household and the AIC level effects, a mixed model repeated measure analysis was employed following the approach presented in the study by Diener and Lucas (23) was employed. For the fixed factors used in both level analyses, categorical variables’ education, household composition, household location, and the three COVID-19-related risk variables were used. Multivariate analysis of variance (MANOVA) was also used to determine the effect of predictors on dependent variables. A pairwise comparison analysis between the levels of independent predictors using Sidak and the LSD (least significant difference) adjustment method was performed and the *p*-values with a false detection rate below 0.05 were considered.

**TABLE 4:** Description of the AIC groups based on local and national COVID-19 restrictions’ impact on households and lockdown working ability: weighted data analysis.

Variable	Level	Very low AIC *N* (%)	Low AIC *N* (%)	High AIC *N* (%)	Very high AIC *N* (%)
Total *N* (%)		715 (100)	1,778 (100)	3,126 (100)	2,381 (100)
1) Travel and movement restrictions	No impact	57 (24.9)	238 (16.2)	507 (17.8)	1,086 (48.0)
	Small impact	65 (28.4)	517 (35.2)	1,286 (45.0)	1,197 (31.1)
	Large impact	107 (46.7)	715 (48.6)	1,062 (37.2)	837 (20.9)
2) Closure or restrictions on public transport	No impact	103 (46.0)	562 (50.3)	1,250 (54.2)	1,282 (61.2)
	Small impact	41 (18.9)	351 (31.5)	637 (27.6)	630 (29.8)
	Large impact	78 (35.1)	203 (18.2)	419 (18.2)	208 (9.0)
3) Closure of restaurants, cafés, and canteens	No impact	57 (32.6)	207 (14.3)	456 (16.4)	576 (24.5)
	Small impact	83 (47.4)	746 (51.3)	1,377 (49.5)	1,613 (49.9)
	Large impact	35 (20.0)	499 (34.4)	949 (34.1)	955 (25.6)
4) Closure of you (physical) workplace	No impact	49 (35.5)	128 (14.3)	324 (17.3)	1,555 (33.7)
	Small impact	20 (14.5)	225 (25.1)	450 (24.0)	391 (30.4)
	Large impact	69 (50.0)	544 (60.6)	1,101 (58.7)	722 (35.9)
5) Closure of education and care institutions	No impact	117 (53.7)	567 (40.1)	1,144 (49.1)	1,555 (64.2)
	Small impact	26 (11.9)	231 (16.3)	418 (18.0)	391 (12.4)
	Large impact	75 (34.4)	616 (43.6)	765 (32.9)	722 (23.4)
6) Closure of other public places	No impact	87 (41.8)	393 (27.9)	853 (33.0)	1,063 (42.4)
	Small impact	55 (26.5)	534 (37.9)	1,047 (40.4)	1,169 (38.6)
	Large impact	66 (31.7)	481 (34.2)	688 (26.6)	612 (19.0)
7) Restrictions on people in one place	No impact	63 (29.2)	286 (19.7)	581 (21.8)	810 (31.7)
	Small impact	77 (35.6)	528 (36.3)	1,224 (45.7)	1,299 (40.0)
	Large impact	76 (35.2)	641 (44.0)	869 (32.6)	920 (28.3)
8) Lockdown working ability	Mean score (*SD*)	0.14 (0.12)	0.40 (1.1)	0.52 (2.0)	0.57 (0.60)

Lockdown working ability is measured from 0.0 as the minimum to 1.0 as the maximum (See text for explanation).

**TABLE 5:** Description of the AIC groups based on national cultural dimensions: weighted data analysis.

Variable	Very low AIC Mean (*SD*)	Low AIC Mean (*SD*)	High AIC Mean (*SD*)	Very high AIC Mean (*SD*)
Power distance	74.7 (113.5)	47.0 (66.3)	45.2 (80.7)	36.9 (66.0)
Individualism	32.3 (36.0)	72.1 (134.1)	82.9 (276.2)	75.2 (70.4)
Masculinity	49.4 (67.7)	66.5 (88.1)	58.8 (217.9)	32.6 (59.1)
Uncertainty avoidance	89.9 (113.1)	75.5 (140.9)	51.0 (142.3)	56.7 (58.0)
Long-term orientation	50.1 (44.5)	59.6 (149.5)	54.1 (138.6)	72.1 (75.6)
Indulgence	31.4 (50.0)	26.9 (73.5)	62.3 (263.9)	58.0 (52.7)

The mean scores are of the scores for each country in a given AIC group. Full explanations for each of the six national cultural dimensions, and how these are derived, are provided in Hofstede Insights (24).

## Results

In this section, the main results are described and commented on in line with the aims of the paper as outlined in section “Aims of this paper.” The overall focus is on the two main food-related behavioral changes of household food purchasing and food consumption, demonstrated in the literature review, and elsewhere, to have been considerably and significantly impacted by COVID-19. Several subject-related predictors are deployed to describe these impacts on both the individual and the AIC levels as also discussed in the literature review, i.e., variable categories represented by education, household composition, residence category, and COVID-19 risk-related variables. As mentioned above, focusing on these specific variables is undertaken to fill an important research gap.

This “Results” section is organized in the following way. First, COVID-19 restrictions, risk perception and cultural profiling across the four AIC groups are described. Next the results of the mixed model analysis are presented, focusing on mixing both the individual household level and the AIC level effects. Finally, a detailed analysis of the changes in the marginal means of food consumption and purchasing during COVID-19, in relation to the AIC groups and household composition categories, is presented.

### Descriptive statistics of actual individual consumption groups in relationship to COVID-19 restrictions, risk perception, and cultural profiling

Table 4 describes the variability of pandemic-induced restrictions and closures across the four AIC groups in rows 1–7. These are as reported, and thus experienced, by household respondents in the survey themselves, which arguably is more likely to influence their behavior than official restrictions. Row 8 provides national data on lockdown working ability during the first wave of COVID-19, obtained from Palomino et al. (19), defined as the capacity of individuals to work under a lockdown which considers their teleworking capacity. The spread of COVID-19 had direct asymmetric effects on the labor market: in principle, only the jobs that can be done from home (“teleworkable”) are unimpeded by the lockdown. Some occupations like health services and food sales are considered essential, so workers are not affected by their capacity to work from home. Meanwhile, certain economic activities like hospitality are closed under the lockdown and working is not at all possible.

Table 4 shows a number of significant trends from the Very Low to the Very High group. Generally, the impact of transport restrictions decreases from the low AIC end to the high AIC end. In terms of closures, the pattern is similar but also more nuanced so that typically the Low group, sometimes together with the High group, sees greater impact than the Very Low group, while the Very High group always experiences least impact except in relation to the closure of restaurants, cafés, and canteens. The possible explanation for the latter is that the Very High group also sees the lowest closure of workplaces and many canteens are part of these workplaces that close less often. In this group, the higher preponderance of white-collar offices as compared to more blue-collar establishments perhaps reflects the nature of the work here as being more easily adaptable to social distancing and other COVID-19 rules. In contrast, the other closures tend to be due to government regulations applied unilaterally rather than on a workplace basis. Overall, it can be seen that the Very High and High groups were both affected less by, and more able to adapt to, pandemic-related restrictions and closures. The existence of this general trend is also shown by the lockdown working ability scores that rise continuously from Very Low to Very High, demonstrating the increased availability and quality of teleworking infrastructures and how conducive to teleworking their occupational profiles are seen to be.

In Table 2, the three types of risk perception, i.e., infection, severity, and anxiety, the perception level generally decreases significantly along the AIC spectrum from Very Low to Very High and is most clearly seen in terms of severity where there is an unbroken progression. Very similar downward trends come from actual household infection, isolation, and hospitalization, where the High AIC group is only a slight outlier to this significant trend.

Table 5 presents an interesting and, as far as we are aware, unique examination of national culture in relation to differences along the AIC dimension, and arguably thereby also in relation to food behavior and changes during COVID-19 as examined in this paper. We have used the Hofstede Insights (24) tool that assigns scores out of 100 for each country across six dimensions of national culture as shown in Table 5.

The national cultural differences across the four AIC groups in Table 5 present some very clear significant trends. Power distance (measuring how far away individuals in a given country feel from the centers of power) shows a marked decline along the Very Low to Very High spectrum. In other words, people toward the higher AIC end tend to feel much more empowered as individuals than their counterparts in the lower AIC countries. A similar trend is seen in terms of uncertainty avoidance, i.e., individuals at the lower AIC end are more likely to attempt to avoid uncertainty in their behavior. The opposite trend of an increasing cultural trait from the low to the high AIC countries is seen in relation to individualism, long-term orientation, and indulgence. The sixth cultural dimension, masculinity, although statistically significant, has a much lower correlation coefficient than the other five and does not appear to vary in a regular manner along the AIC spectrum, although it might be interesting to note that the Very High AIC group has the lowest masculinity score.

### Modeling analysis

The repeated mixed model analysis, mixing both individual household and AIC levels, due to the effects of the first wave of the COVID-19 pandemic, was conducted to describe the relationship between selected predictors and the dependent variables of food consumption and food purchasing. The two models at both levels include the same predictors, education, household composition, household location, and perceived risk of infection, severity, and anxiety, with the second level additionally analyzing the effect of AIC itself as a predictor.

Results for the models describing changes in consumption and purchasing due to the COVID-19 pandemic are presented in Table 6. The models explain food consumption and purchasing change in the behavior at both the individual and AIC levels. General consumption changes increase on average by.014 (–0.019;0.046), while purchasing change decreases by.270 (–0,321; –0,219). Both consumption and purchasing change vary significantly at the individual and AIC levels. The results in terms of the association between predictors and outcomes for food consumption show significant variation between the categories of education, risk of infection, and severity at the individual household level, while household composition and risk of infection vary significantly at the AIC level. In terms of purchasing change, significant variation was observed for all predictors on both levels.

**TABLE 6:** Repeated measures mixed-model analysis with individual household and AIC levels of regional, household composition, educational, and COVID-19 risk perception effects on change in consumption and purchasing of food due to effects of the COVID-19 pandemic.

Model	Individual (Level 1)	AIC (Level 2)	df*	*F*	Sig.
**1. Food consumption**					
Intercept			1;65539	0.70	0.404
	Household composition		2;65539	1.40	0.246
		Household composition	6;65539	3.30	0.003
	Education		2;65539	4.54	0.011
		Education	6;65539	1.97	0.066
	Household location		2;65539	0.18	0.835
		Household location	6;65539	1.24	0.283
	Risk for infection		2;65539	8.0	<0.001
		Risk for infection	6;65539	5.98	<0.001
	Risk for severity		2;65539	11.2	<0.001
		Risk for severity	6;65539	0.48	0.835
	Risk for anxiety		2;65539	0.40	0.671
		Risk for anxiety	6;65539	0.74	0.621
**2. Food purchasing**					
Intercept			1;24031	108.5	<0.001
	Household composition		2;24031	3.59	0.028
		Household composition	6;24031	20.9	<0.001
	Education		2;24031	10.54	0.01
		Education	6;24031	5.13	<0.001
	Household location		2;24031	7.22	0.001
		Household location	6;6010	2.19	0.041
	Risk for infection		2;24031	5.65	0.004
		Risk for infection	6;24031	7.28	<0.001
	Risk for severity		2;24031	3.32	0.036
		Risk for severity	6;24031	3.2	0.004
	Risk for anxiety		2;24031	66.6	<0.001
		Risk for anxiety	6;24031	7.0	<0.001

*Cells values in the column (df) represent the degrees of freedom for numerator and denominator.

In the between-subject analysis, using the pairwise comparison tests on the predictor levels’ marginal means at the individual household level, we detect several mean change differences in each of the two main dependent variables (Table 7). For both food consumption and purchasing, the variables of education, risk of infection, and severity showed significant differences, while the variables of household composition and risk for anxiety were only significantly different for food purchasing change. The lower education category had the largest increase in food consumption and the largest decrease in food purchasing and was significantly or notably different from the remaining two categories. People living in single-person households experienced the lowest decrease in food purchasing, which is significantly different from people living in a household with children aged 0–19. Significant differences were also observed between different categories of risk of infection and risk of severity for both dependent variables, while the categories of risk of anxiety were only significantly different for food purchasing.

**TABLE 7:** Model marginal means of different individual household level effects on change in consumption and purchasing food during the COVID-19 pandemic.

Predictor variables	Category	Mean consumption change	Mean purchasing change
		(During-before COVID-19)	(During-before COVID-19)
Education	Lower secondary or equivalent	0.058 (–0.036; 0.153)^ab^	–0.100 (–0.249; –0.049)^a^
	Upper secondary of equivalent	–0.019 (–0.032; –0.006)^a^	–0.336 (–0.357; –0.316)^b^
	University degree or equivalent	–0.002 (–0.009; 0.013)^b^	–0.374 (–0.391; –0.357)^c^
Household composition	Household with children 0–19	0.007 (–0.026; –0.041)	–0.293 (–0.346; –0.240)^a^
	Single-person household	0.013 (–0.023; –0.048)	–0.248 (–0.304; –0.192)^b^
	Households with 2 + adults without children	0.021 (–0.012; 0.054)	–0.269 (–0.321; –0.217)^ab^
Household location	Urban	0.012 (–0.021; –0.045)	–0.243 (–0.295; –0.191)^a^
	Intermediate	0.013 (–0.021; 0.047)	–0.271 (–0.325; –0.218)^b^
	Rural	0.017 (–0.017; 0.051)	–0.296 (–0.350; –0.241)^b^
Risk infection	Low	–0.010 (–0.044; 0.024)^a^	–0.301 (–0.355; –0.248)^a^
	Medium	0.019 (–0.015; 0.053)^b^	–0.261 (–0.315; –0.208)^b^
	High	0.032 (–0.003; 0.068)^b^	–0.247 (–0.315; –0.208)^b^
Risk severity	Low	0.041 (0.0’6; 0.075)^a^	–0.249 (–0.303; –0.195)^a^
	Medium	0.011 (–0.045; 0.024)^b^	–0.268 (–0.322; –0.215)^ab^
	High	–0.011 (–0.045; 0.024)^c^	–0.293 (–0.347; –0.238)^b^
Risk anxiety	Low	0.019 (–0.016; 0.053)	–0.168 (–0.222; –0.114)^a^
	Medium	0.010 (–0.024; –0.044)	–0.275 (–0.328; –0.222)^b^
	High	0.013 (–0.022; 0.047)	–0.367 (–0.421; –0.313)^c^

Based on individual fixed level estimated marginal means. Higher absolute values mean bigger change. Positive signs mean increased consumption/purchasing as affected by COVID-1919, while negative signs denote decreases. Data weighted by countries. The mean differences are significant at the 0.05 level. Different superscript letters indicate differences between groups. Adjustment for multiple comparisons was conducted using the LSD method.

Tables 8, 9 present the model post-estimation means for different categories within the AIC and individual household levels for all predictors for changes in consumption and purchasing of food due to COVID-19.

**TABLE 8:** Model post-estimates means (*SD*) for different AIC and individual household level effects describing the change in consumption of food during the COVID-19 pandemic.

Variables	Very low AIC	Low AIC	High AIC	Very high AIC
**Education**				
Lower secondary or equivalent	–0.13 (0.05)	0.41 (0.00)	–0.06 (0.03)	–0.02 (0.02)
Upper secondary of equivalent	–0.06 (0.05)	–0.01 (0.03)	–0.01 (0.03)	0.00 (0.02)
University degree or equivalent	–0.03 (0.05)	0.01 (0.03)	0.02 (0.04)	0.02 (0.03)
**Household composition**				
Households with children 0–19	–0.10 (0.04)	0.00 (0.04)	0.01 (0.04)	0.04 (0.02)
Single-person households	–0.02 (0.04)	–0.01 (0.03)	–0.01 (0.04)	0.00 (0.02)
Households with two or more adults without children	–0.03 (0.05)	0.00 (0.03)	0.02 (0.04)	–0.01 (0.02)
**Household location**				
Urban	–0.03 (0.05)	–0.01 (0.04)	0.02 (0.04)	0.01 (0.03)
Intermediate	–0.05 (0.06)	0.02 (0.03)	–0.01 (0.04)	0.01 (0.03)
Rural	–0.06 (0.06)	0.01 (0.03)	0.02 (0.04)	–0.01 (0.03)
**Risk of infection**				
Low	–0.07 (0.05)	–0.01 (0.03)	0.00 (0.04)	0.01 (0.02)
Medium	–0.07 (0.04)	0.02 (0.02)	0.01 (0.04)	0.01 (0.02)
High	0.02 (0.04)	–0.03 (0.06)	0.04 (0.04)	–0.03 (0.03)
**Risk of severity**				
Low	–0.04 (0.05)	0.01 (0.03)	0.03 (0.04)	0.02 (0.02)
Medium	–0.06 (0.05)	0.01 (0.04)	0.00 (0.03)	0.01 (0.02)
High	–0.04 (0.06)	–0.02 (0.03)	0.00 (0.04)	–0.03 (0.02)
**Risk of anxiety**				
Low	–0.05 (0.05)	0.00 (0.04)	0.03 (0.04)	0.02 (0.02)
Medium	–0.05 (0.06)	0.00 (0.03)	0.00 (0.04)	0.00 (0.02)
High	–0.04 (0.06)	0.00 (0.04)	0.01 (0.04)	–0.01 (0.03)

**TABLE 9:** Model post-estimates means (*SD*) for different AIC and individual household level effects describing the change in purchasing of food during the COVID-19 pandemic.

Variables	Very low AIC	Low AIC	High AIC	Very high AIC
**Education**				
Lower secondary or equivalent	–0.34 (0.10)	0.63 (0.18)	–0.39 (0.11)	–0.22 (0.11)
Upper secondary of equivalent	–0.51 (0.15)	–0.19 (0.14)	–0.38 (0.11)	–0.24 (0.11)
University degree or equivalent	–0.41 (0.13)	–0.29 (0.14)	–0.40 (0.11)	–0.28 (0.10)
**Household composition**				
Households with children 0–19	–0.69 (0.08)	–0.17 (0.14)	–0.42 (0.10)	–0.23 (0.10)
Single-person households	–0.34 (0.08)	–0.35 (0.13)	–0.36 (0.10)	–0.21 (0.10)
Households with two or more adults without children	–0.40 (0.09)	–0.30 (0.13)	–0.40 (0.11)	–0.28 (0.11)
**Household location**				
Urban	–0.39 (0.12)	–0.24 (0.16)	–0.35 (0.09)	–0.25 (0.11)
Intermediate	–0.47 (0.15)	–0.23 (0.15)	–0.40 (0.11)	–0.27 (0.10)
Rural	–0.53 (0.15)	–0.29 (0.15)	–0.43 (0.10)	–0.23 (0.11)
**Risk of infection**				
Low	–0.51 (0.15)	–0.22 (0.15)	–0.39 (0.10)	–0.20 (0.08)
Medium	–0.47 (0.14)	–0.23 (0.13)	–0.41 (0.11)	–0.28 (0.09)
High	–0.36 (0.10)	–0.36 (0.17)	–0.39 (0.11)	–0.38 (0.11)
**Risk of severity**				
Low	–0.46 (0.15)	–0.18 (0.16)	–0.35 (0.08)	–0.19 (0.07)
Medium	–0.46 (0.15)	–0.28 (0.15)	–0.38 (0.10)	–0.26 (0.08)
High	–0.45 (0.15)	–0.28 (0.13)	–0.46 (0.11)	–0.38 (0.10)
**Risk of anxiety**				
Low	–0.44 (0.15)	–0.08 (0.11)	–0.31 (0.05)	–0.18 (0.05)
Medium	–0.49 (0.15)	–0.27 (0.11)	–0.39 (0.06)	–0.26 (0.05)
High	–0.43 (0.15)	–0.35 (0.11)	–0.54 (0.07)	–0.42 (0.06)

The results in Table 8 show the highest mean decrease in consumption of food for subjects with lower secondary education, especially in the Very Low AIC group. There is a gradual decrease in the mean change of food consumption from the Very High to the Very Low AIC group in all household composition categories. Households with children located in the Very Low AIC group show the highest decrease in consumption of all categories. In terms of household location, we observed a lower decrease in consumption change moving from Very Low to Very High AIC, with subjects living in urban locations generally having the lowest decrease in consumption change. Increasing the category of risk for COVID-19 infection increases the change in consumption of food for subjects located in the Very High AIC group, with those in the high-risk category showing the highest mean decreased change. Conversely, subjects located in the Very Low AIC group increase their consumption of food, thereby increasing the category of risk for infection.

In terms of purchasing changes for subject-related factors within different AIC groups shown in Table 9, we observed a clear decrease in change from Very Low to Very High AIC in almost all observed variables and corresponding levels. Subjects living in rural areas and those living in households with children showed the highest decrease in change of food purchasing in the Very Low AIC group, while no such trend was observed in the Very High AIC group.

### Actual individual consumption and household composition model estimates for change in consumption and purchasing on a food categories’ level

**FIGURE 2:**
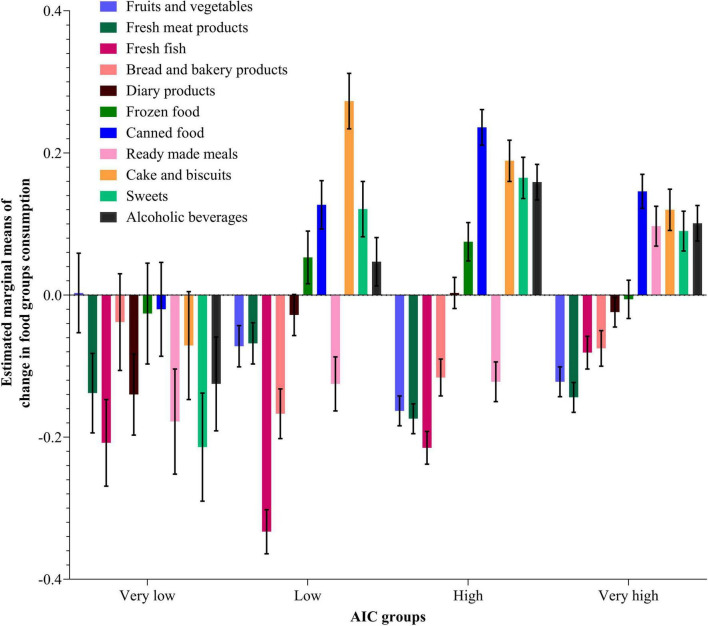
Estimated marginal means using the MANOVA procedure for different food types consumption change (During—Before COVID-19) per AIC groups. Data weighted by countries (see also Supplementary Tables 2, 4).

**FIGURE 3:**
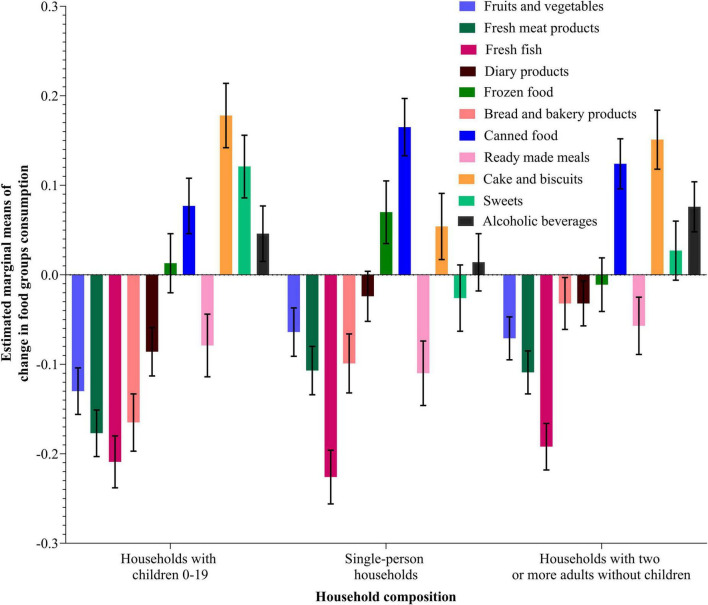
Estimated marginal means using the MANOVA procedure for different food types consumption change (During—Before COVID-19) per household composition categories. Data weighted by countries (see also Supplementary Table 3).

Figures 2, 3 show the estimated marginal means and standard errors of consumption changes for 11 food types at the AIC level and per household composition, respectively. The results of the pairwise comparison analysis between different AIC groups and household composition categories for the consumption of different food types affected by COVID-19 show many significant differences between the analyzed types. Regarding the AIC groups, significant differences were observed in fresh meat consumption for both High and Low AIC groups (*p* = 0.001); fresh fish between the Low, High, and Very High groups; bread and bakery products between the Low and Very High groups; frozen food between the High and Very High groups (*p* = < 0.05); between all AIC groups for canned food; between the Very High group and all other groups for readymade meals; and between different groups of AIC for cake and biscuits, sweets, and alcoholic beverages consumption. Different categories of household composition were significantly different for fruits and vegetables, meat and meat products, bread and bakery products, dairy products, frozen food, cake and biscuits, and sweets.

**FIGURE 4:**
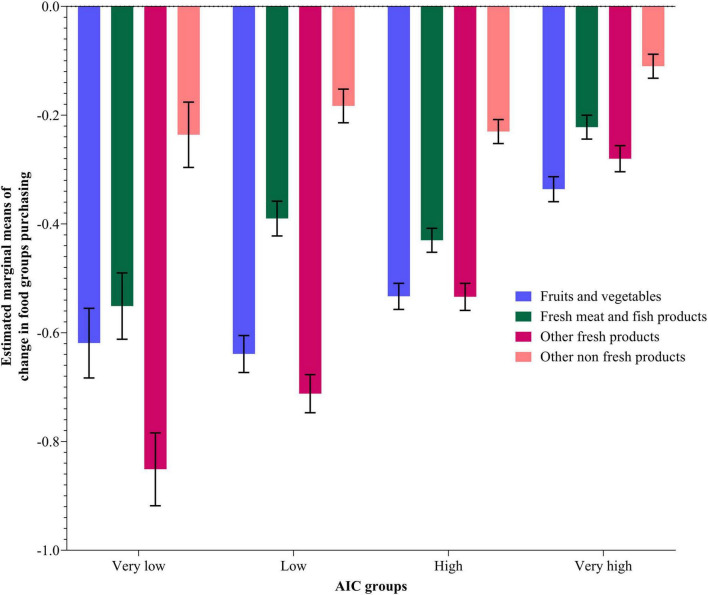
Estimated marginal means using MANOVA procedure for different food types purchasing change (During—Before COVID-19) per AIC groups. Data weighted by countries (see also Supplementary Table 5).

**FIGURE 5:**
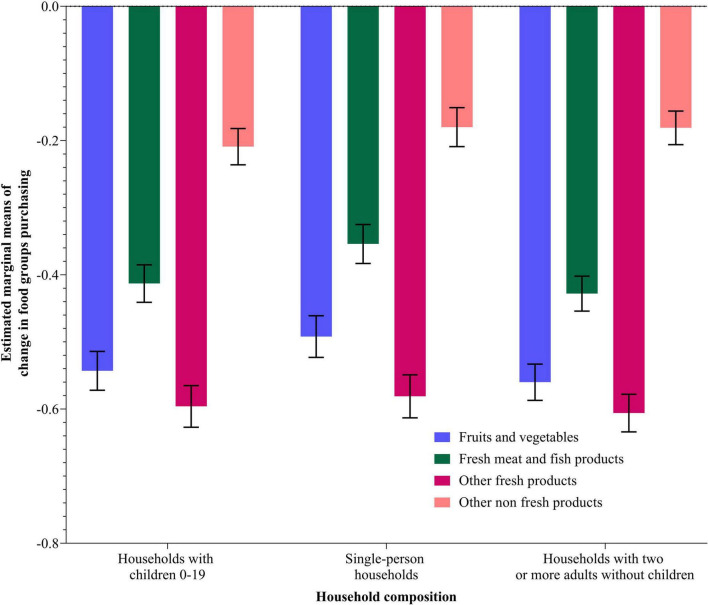
Estimated marginal means using MANOVA procedure for different food types purchasing change (During—Before COVID-19) per household composition categories. Data weighted by countries (see also Supplementary Table 3).

Figures 4, 5 present the estimated marginal means and standard errors of purchasing changes for the four food types per AIC and household composition, respectively. The pairwise comparison analysis of different AIC groups and household composition categories for different food purchasing types shows significant differences. Significant differences were observed between all AIC groups in fruit and vegetables, meat and meat products, and other fresh food products purchasing change affected by COVID-19. For the other non-fresh food products, the Very High AIC group was significantly different from all other groups except from the Very Low AIC group. In the household composition groups, significant differences are observed between all levels within the fruits and vegetables purchasing type, within meat and meat products, within other fresh and non-fresh food types, and between single-person households and the other two household composition categories.

## Discussion

This paper has attempted to focus on the most likely predictor and outcome variables that can help explain food behavior changes during COVID-19. The results presented in the “Results” section are striking and show that the measure of financial status we have deployed, i.e. national AIC as the main indirect predictor, and household composition as the main direct predictor, provide powerful statistically significant explanations of behavioral changes in household food consumption and purchasing. We have also examined other predictors that contribute explanatory power to the food behavioral changes seen during the first wave of COVID-19.

### Actual individual consumption’s effect on food behavior changes during COVID-19

There are clear statistically significant differences between the four examined AIC groups. In terms of pandemic-related regulatory restrictions and closures, the Very High and High AIC groups were both affected less by, and more able to adapt to, such regulations. The existence of this general trend is also shown by the lockdown working ability scores that rise continuously from Very Low to Very High, demonstrating the increased availability and quality of teleworking infrastructures and how conducive to teleworking their occupational profiles are seen to be. This trend of increasing resilience of households from the low AIC end to the high end is underlined by a strong decrease in the three types of risk perception that households report, i.e., infection, severity, and anxiety, especially in terms of severity. Very similar decreasing trends are seen in the actual household COVID-19 experiences of infection, isolation, and hospitalization.

Other predictors examined along the AIC dimension include national cultural differences that also reveal significant regular changes. Both power distance and uncertainty avoidance decrease from the low AIC end to the high end, while the three cultural traits of individualism, long-term orientation, and indulgence increase toward the high end. This paints a clear picture of cultural differences, which arguably reveals quite different mindsets and worldviews that are likely to influence how individuals react to severe shocks like the COVID-19 pandemic. Examining these national cultural scores is an exercise to see whether they might offer some insight into understanding how and why different countries were affected by and reacted to the pandemic in different ways. It is clear that there are many relatively strong similarities between culture and AIC, although this by no means implies any causation between the two, and there are likely to be complex explanations and other intervening variables that would need to be considered. This is beyond the scope of this paper but might be taken up in further research.

### Individual household and actual individual consumption-related effects on food consumption and food purchasing change

When looking specifically at the contributions of Level 1 individual household and Level 2 AIC predictors in explaining the changes in food consumption and purchasing during COVID-19, education, household composition, and risk of infection were the most powerful or joint most powerful predictors examined in both tested models. Looking at the food consumption results, the modeling analysis in the “Modeling analysis” section showed that both the perceived COVID-19 risks of infection and education are significant or notably significant (*p* < 0.1) predictors at both the individual household and AIC levels, while the risk of COVID-19 severity was only significant at the individual household level, and household composition was only significant at the AIC level analysis. The food purchasing model shows higher exploratory power, with all predictors being significant in both individual household and AIC level analyses.

Leaving aside the power of AIC’s monetary measure of consumption as an important aspect of a household’s financial situation for both food consumption and purchasing, it is clear that changes in food consumption and food purchasing behavior are explained by different predictor mixes. There are a number of possible reasons for this especially, but not only, during a crisis. First, households may be forced to purchase food items that are actually available when they shop rather than items they would normally buy but cannot due to non-availability. The immense supply chain delays, shortages, and other restrictions have obviously created such constraints. Second, many people grow at least some of their food rather than purchase it, and this increased significantly during the crisis by about 25% in rural areas, where there is often more space, and about 10% in urban areas (2). In this context, some households are able to secure items for consumption, temporarily not available in the shops, from family or friends who do grow their own food or who have been able to stock up on specific items to share.

Turning to the specific predictor mixes in the two types of food behavior, food consumption tends to be strongly associated by each individual with their physical health, so the level of education about this link is important, as is the perceived risk of COVID-19 infection and severity. Putting food into one’s body, especially during a serious pandemic, is likely to be seen as something to be taken extremely seriously. The type of household composition seems to be less powerful in this context, except perhaps when related to sensitivity about these issues where children or older persons are present in a household.

In contrast, food purchasing is much more constrained by the regulatory context of restrictions and closures in terms of where, when, and how often food shopping is possible and what is available on a given day. Thus, at the individual household level, all three COVID-19-related risk factors were confirmed as powerful predictors of food purchasing, unlike with food consumption where only risk of infection and severity were detected as significant. Additionally, where a given household is located, which is directly related to the regulatory environment and food supply, and thereby what food can be purchased, was also found important. Because of haphazard food availability during a crisis, location is also likely to affect the stocking up of food, which increased by over 50% during COVID-19 in urban areas and by about 30% in rural areas, as did lockdown restrictions and the incidence of COVID-19 infections (2). For purchasing, the type of household composition is a significant predictor at both levels, individual household and AIC, compared to food consumption, given that this helps to determine the amount and range of foodstuffs acquired, whether eventually eaten or not. In households with children, there are typically more mouths to feed and, thus, more differences in food tastes to accommodate, so stocking up is also likely to be more important than for other households, especially in the context of relatively constrained shopping opportunities. These conclusions are also strengthened through the analysis of different AIC groups, which appear to be significantly associated with the change due to the COVID-19 effects on both food purchasing and consumption.

### Actual individual consumption’s effect on food consumption and food purchasing change on a food category level

Looking along the AIC dimension on its own, the Very Low AIC group had no increases in any type of food consumption measured in the survey and large decreases in most foods. In comparison, the Low group had higher decreases in fresh fish and bakery products than the Very Low group, but had increases in all processed foods (frozen and canned foods) and all “comfort” foods (cake, biscuits, sweets, and alcohol). On the one hand, this seems to indicate the greater financial strain on Very Low AIC households, resulting in reduced consumption of all food types measured in the survey. On the other hand, the Low AIC group, although still relatively financially strained, was nevertheless able to indulge in some increase in processed foodstuffs and very high increases in comfort foods, possibly due to some stress during the lockdown, as well as because such foods are normally cheaper than fresh foods and have longer shelf lives. In terms of the frequency of food purchasing, although all AIC groups saw only decreases, these were the greatest in the Very Low group and only slightly less large in the Low AIC group.

People living in countries in the Very High AIC group experienced the lowest decreases in fresh food consumption, as well as modest increases in processed and comfort foods. In terms of the frequency of food purchasing, although this decreased across all groups and in all food types during the pandemic due to restrictive shopping possibilities, the Very High AIC group also had the lowest decrease. These households seem to have suffered much less from financial strain than the other three groups, although still subject to some, probably non-financial, stress by increasing comfort food consumption which was probably already at a relatively high level. The High Group shows similar patterns to the Very High group but with somewhat greater change, i.e., larger decreases in fresh food consumption (though not as much decrease as the Low group), and larger increases in processed and comfort food consumption. Similarly, the High Group saw smaller decreases in food purchasing than the two low groups but larger than the Very High group. Thus, the High group seems to be quite similar to the Very High group but simultaneously shares more of the characteristics of the Low group. This again underlines the view that AIC reflects important aspects of a household’s financial situation and that the higher AIC groups are more financially resilient, less subject to stress, and thereby also more able to withstand the food shock of COVID-19.

### Household composition effect on food consumption and food purchasing change on a food type level

Some similar conclusions can be drawn about household composition as a predictor where some household types seem generally more resilient than others. For example, households with children had the highest decreases in food consumption across all food types, except bakery products, as well as the highest overall decrease in the frequency of food purchasing. Households with children are significantly different in their food behavior changes compared to the other two categories of households. Having children in the household is clearly a factor that increases the likelihood of changes in household food behavior during an economic shock, probably because they are more likely to be financially vulnerable as their incomes have to feed more mouths. Parents are also more likely to be concerned about the health aspects of food intake for children, especially during a pandemic, and whether they are financially able to act on this concern. In terms of food purchasing, households with two or more adults without children also saw large decreases, indeed slightly more than households with children in terms of fresh fruit and vegetables and other fresh food products. Perhaps, this reflects the lower concern in households without children as they have to eat fresh food given that they have the highest mean ages and that, while households with children are more likely to be concerned to eat fresh food, they are much more financially stretched. Single-person households show the lowest decreases in the frequency of purchase, perhaps because these households tend to be younger than other households and are, thus, less COVID-19-anxious, so they are engaged in a relatively more frequent shopping. In particular, these generally more youthful households are more likely to be food-aware, and thus continue purchasing and consuming as much fresh fruit and vegetables as possible.

## Conclusion

The above observations and conclusions demonstrate the markedly different characteristics of individual households represented through the selected variables and within the four AIC groups. Other variables not considered in this paper would undoubtedly provide additional evidence, demonstrating the complexity in attempting to untangle and explain food-related COVID-19-induced behavioral changes. In this paper, we have attempted to justify our selection of the specific variables we have focused on, based on the extant literature provided through our research. However, this is constantly open to constructive criticism and improvement as our knowledge of how and why food-related behavioral change takes place.

Most of the food behavior changes charted in this paper can be interpreted as relatively negative in terms of the nutritional value of food, for example in the large decreases in fresh food consumption alongside the large increases in both processed food and comfort food products. This is perhaps unsurprising given the massive economic constraints the pandemic occasioned and the consequential social damage caused. These arguably portend the likely outcomes of any other future shocks and crises that will probably arise, whether these be further threats to health, economic disruptions due to macro-economic and political conditions, and/or to environmental degradation and stress. Indeed, it is already the case that these and other crises are intrinsically interrelated (25).

This paper attempts to contribute to food behavior research in the context of COVID-19 as a severe socio-economic shock and to assist in pinpointing potential weak points in existing food systems and broader policies that should be addressed given the likelihood of similar future shocks. At least in the European context, but arguably also more widely and without at all dismissing important national variations, it is clear from this paper that the main predictors of negative food behavior change, and thus, the main weak points in the present system that need to be addressed, are the following:

• Of first rank importance is the need to support households’ financial resilience, especially for those already financially strained.
• The importance of ensuring that different categories of households are addressed in relation to their specific needs (whether with or without children and the household’s age spectrum), thereby, eschewing a one-size-fits-all approach.
• Communicating and supporting transparent messaging and policies to raise awareness of particular food and health issues both during a crisis, as well as more generally, and to mitigate the anxiety and risk stresses that any crisis throws up. Behavioral science approaches are needed; for example, that provide suitable “nudges” making it easier for individuals and households to make good decisions about healthy food and diets. This also needs to be recognized that there are educational and awareness differences in different population cohorts and locations.
• Recognizing the importance of place and where households live, especially the significant differences and needs of urban and rural locations.

This paper also demonstrates the differential effects of lockdowns, restrictions, and closures on how food behavior changes, as well as the clear relationship between national cultural traits and financial resilience, although more research should focus on these issues. Clearly, strengthening and increasing the resilience of both health and food systems as critical sectors of the economy also require high-priority consideration, but these issues have not been directly addressed in this paper.

## Data availability statement

The original contributions presented in this study are included in the article/Supplementary material, further inquiries can be directed to the corresponding author.

## Author contributions

HH devised the overall focus and approach of the manuscript and undertook the data preparation and statistical analyses. JM undertook the main writing and interpretation of the results, supported by HH and IP. IP and MJ provided critical reviews and comments. All authors, together with all organizations listed in the Acknowledgment section, prepared the dataset used for this article, while HH prepared additional variables needed specifically for the manuscript.

## Footnotes

1
https://www.food-COVID-19.org/
