# Supplementary material for: European household spending and socio-economic impacts on food behavior during the first wave of COVID-19
Source: Front Nutr. 2022 Aug 3;9:869091. doi: 10.3389/fnut.2022.869091 (PMC9382126; doi:10.3389/fnut.2022.869091)
Supplement: Supplementary file 1 [file Data_Sheet_1.docx]

# Supplementary Material

Supplementary Figure 1. Mean change in consumption of different food categories in relationship to mean AIC per head & PPPs of 2020. Data weighted by countries.


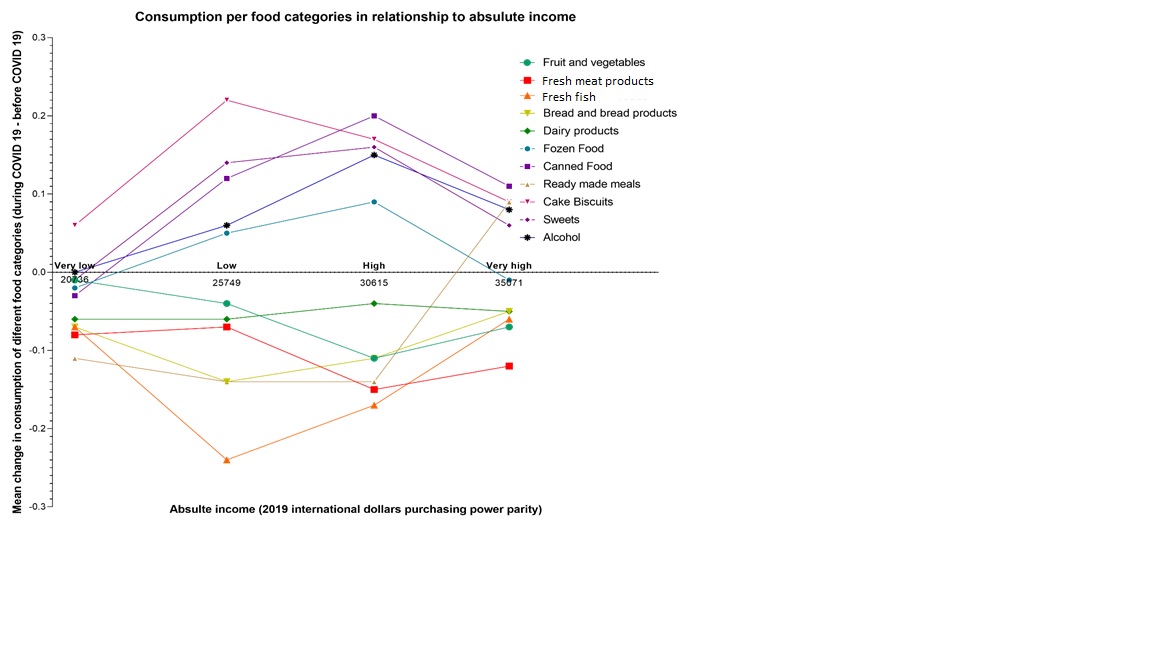


Supplementary Figure 2. Mean change in purchasing of different food categories in relationship to mean AIC per head & PPPs of 2020. Data weighted by countries.


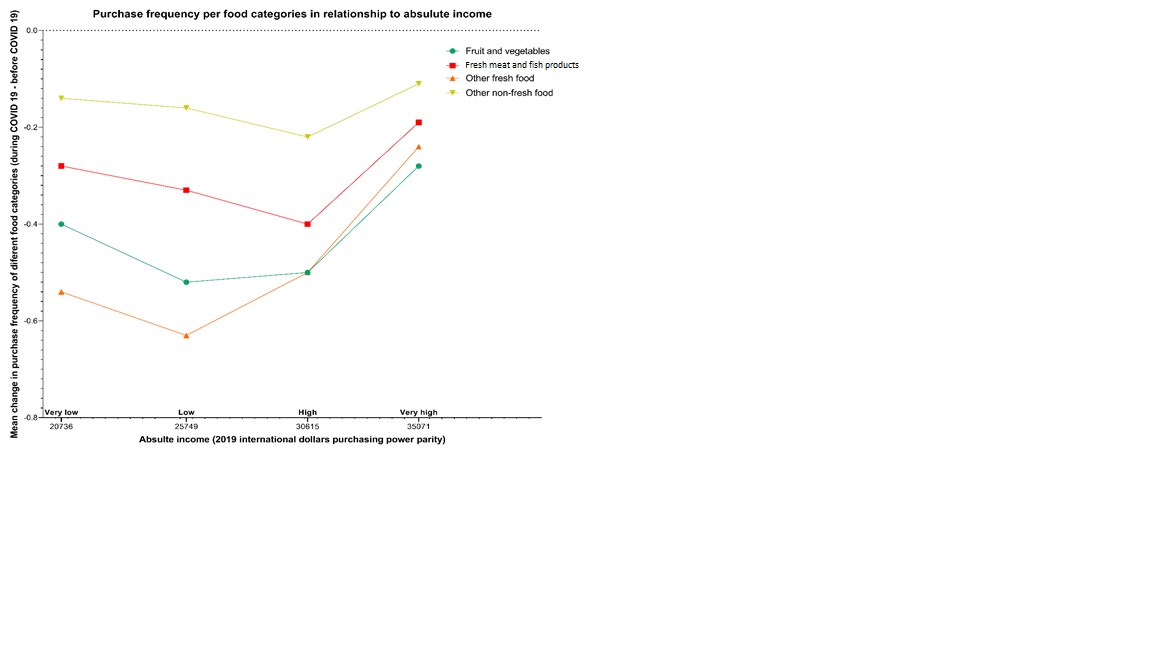


Supplementary Table 1. Corelation between COVID 19 affected consumption change of different food categories per AIC groups

|  | Fruit and vegetables | Meat and meat products | Fish and fish products | Bread and bread products | Dairy products | Frozen Food | Canned Food | Ready made meals | Cake Biscuits | Sweets | Alcohol |
| --- | --- | --- | --- | --- | --- | --- | --- | --- | --- | --- | --- |
| AIC group 1 | | | | | | | | | | | |
| Fruit and vegetables | 1.000 | 0.184** | 0.191** | 0.129** | 0.140** | 0.001 | -0.080* | -0.034 | 0.008 | 0.092* | -0.024 |
| Meat and meat products | 0.184** | 1.000 | 0.178** | 0.250** | 0.191** | 0.011 | -0.055 | 0.020 | 0.088* | 0.132** | 0.043 |
| Fish and fish products | 0.191** | 0.178** | 1.000 | 0.091 | 0.083* | -0.011 | -0.013 | 0.032 | 0.011 | 0.023 | -0.031 |
| Bread and bread products | 0.129** | 0.250** | 0.091 | 1.000 | 0.275** | 0.058 | 0.101* | -0.051 | 0.052 | 0.125** | 0.094* |
| Dairy products | 0.140** | 0.191** | 0.083* | 0.275** | 1.000 | 0.096* | 0.046 | 0.010 | 0.117** | 0.195** | 0.036 |
| Frozen Food | 0.001 | 0.011 | -0.011 | 0.058 | 0.096* | 1.000 | 0.276** | 0.116** | 0.104** | 0.117** | 0.109** |
| Canned Food | -0.080* | -0.055 | -0.013 | 0.101* | 0.046 | 0.276** | 1.000 | 0.186** | 0.047 | 0.088* | 0.135** |
| Ready-made meals | -0.034 | 0.020 | 0.032 | -0.051 | 0.010 | 0.116** | 0.186** | 1.000 | 0.208** | 0.175** | 0.210** |
| Cake Biscuits | 0.008 | 0.088* | 0.011 | 0.052 | 0.117** | 0.104** | 0.047 | 0.208** | 1.000 | 0.461** | 0.235** |
| Sweets | 0.092* | 0.132** | 0.023 | 0.125** | 0.195** | 0.117** | 0.088* | 0.175** | 0.461** | 1.000 | 0.328** |
| Alcohol beverages | -0.024 | 0.043 | -0.031 | 0.094* | 0.036 | 0.109** | 0.135** | 0.210** | 0.235** | 0.328** | 1.000 |
| AIC group 2 | | | | | | | | | | | |
| Fruit and vegetables | 1.000 | 0.186** | 0.161** | 0.181** | 0.167** | -0.066** | -0.136** | -0.101** | 0.037 | 0.028 | 0.025 |
| Meat and meat products | 0.186** | 1.000 | 0.232** | 0.166** | 0.159** | 0.005 | -0.015 | -0.111** | -0.032 | 0.020 | 0.024 |
| Fish and fish products | 0.161** | 0.232** | 1.000 | 0.140** | 0.060* | -0.040 | -0.036 | -0.003 | -0.083** | 0.002 | 0.066** |
| Bread and bread products | 0.181** | 0.166** | 0.140** | 1.000 | 0.297** | -0.042 | -0.055* | -0.013 | 0.112** | 0.095** | 0.085** |
| Dairy products | 0.167** | 0.159** | 0.060* | 0.297** | 1.000 | 0.034 | -0.004 | -0.136** | 0.078** | 0.063** | 0.073** |
| Frozen Food | -0.066** | 0.005 | -0.040 | -0.042 | 0.034 | 1.000 | 0.344** | 0.042 | -0.015 | 0.023 | 0.005 |
| Canned Food | -0.136** | -0.015 | -0.036 | -0.055* | -0.004 | 0.344** | 1.000 | 0.163** | 0.011 | 0.023 | -0.016 |
| Ready-made meals | -0.101** | -0.111** | -0.003 | -0.013 | -.136** | 0.042 | 0.163** | 1.000 | -0.056* | 0.003 | -0.007 |
| Cake Biscuits | 0.037 | -0.032 | -0.083** | 0.112** | 0.078** | -0.015 | 0.011 | -0.056* | 1.000 | 0.349** | 0.174** |
| Sweets | 0.028 | 0.020 | 0.002 | 0.095** | 0.063** | 0.023 | 0.023 | 0.003 | 0.349** | 1.000 | 0.175** |
| Alcohol beverages | 0.025 | 0.024 | 0.066** | 0.085** | 0.073** | 0.005 | -0.016 | -0.007 | 0.174** | 0.175** | 1.000 |
| AIC group 3 | | | | | | | | | | | |
| Fruit and vegetables | 1.000 | 0.384** | 0.296** | 0.189** | 0.190** | -.136** | -0.111** | -0.068** | 0.007 | 0.043* | 0.056** |
| Meat and meat products | 0.384** | 1.000 | 0.411** | 0.177** | 0.204** | -.040* | -0.046** | 0.045* | 0.085** | 0.120** | 0.120** |
| Fish and fish products | 0.296** | 0.411** | 1.000 | 0.139** | 0.062** | -.039* | -0.059** | 0.056** | 0.067** | 0.062** | 0.075** |
| Bread and bread products | 0.189** | 0.177** | 0.139** | 1.000 | 0.152** | -0.032 | -0.008 | 0.039* | 0.122** | 0.095** | 0.106** |
| Dairy products | 0.190** | 0.204** | 0.062** | 0.152** | 1.000 | 0.029 | 0.049** | 0.056** | 0.067** | 0.103** | 0.056** |
| Frozen Food | -0.136** | -0.040* | -0.039* | -0.032 | 0.029 | 1.000 | 0.474** | 0.127** | 0.069** | 0.055** | 0.036* |
| Canned Food | -0.111** | -0.046** | -0.059** | -0.008 | 0.049** | 0.474** | 1.000 | 0.118** | 0.094** | 0.021 | 0.097** |
| Ready-made meals | -0.068** | 0.045* | 0.056** | 0.039* | 0.056** | 0.127** | 0.118** | 1.000 | 0.131** | 0.121** | 0.041* |
| Cake Biscuits | 0.007 | 0.085** | 0.067** | 0.122** | 0.067** | 0.069** | 0.094** | 0.131** | 1.000 | 0.528** | 0.199** |
| Sweets | 0.043* | 0.120** | 0.062** | 0.095** | 0.103** | 0.055** | 0.021 | 0.121** | 0.528** | 1.000 | 0.202** |
| Alcohol beverages | 0.056** | 0.120** | 0.075** | 0.106** | 0.056** | 0.036* | 0.097** | 0.041* | 0.199** | 0.202** | 1.000 |
| AIC group 4 | | | | | | | | | | | |
| Fruit and vegetables | 1.000 | 0.191** | 0.118** | 0.219** | 0.173** | -0.039 | -0.009 | -0.089** | 0.064** | -0.050* | -0.015 |
| Meat and meat products | 0.191** | 1.000 | 0.114** | 0.138** | 0.164** | -0.002 | 0.002 | -0.062** | -0.003 | 0.012 | -0.014 |
| Fish and fish products | 0.118** | 0.114** | 1.000 | 0.008 | 0.032 | -0.009 | 0.010 | -0.010 | 0.058** | 0.014 | -0.002 |
| Bread and bread products | 0.219** | 0.138** | 0.008 | 1.000 | 0.273** | 0.039 | 0.076** | 0.057** | 0.033 | -0.001 | 0.002 |
| Dairy products | 0.0173** | 0.164** | 0.032 | 0.273** | 1.000 | 0.185** | 0.079** | -0.008 | 0.111** | 0.017 | 0.038 |
| Frozen Food | -0.039 | -0.002 | -0.009 | 0.039 | 0.185** | 1.000 | 0.343** | 0.256** | 0.185** | 0.105** | 0.121** |
| Canned Food | -0.009 | 0.002 | 0.010 | 0.076** | 0.079** | 0.343** | 1.000 | 0.297** | 0.135** | 0.135** | 0.106** |
| Ready-made meals | -0.089** | -0.062** | -0.010 | 0.057** | -0.008 | 0.256** | 0.297** | 1.000 | 0.104** | 0.140** | 0.126** |
| Cake Biscuits | 0.064** | -0.003 | 0.058** | 0.033 | 0.111** | 0.185** | 0.135** | 0.104** | 1.000 | 0.360** | 0.190** |
| Sweets | -0.050* | 0.012 | 0.014 | -0.001 | 0.017 | 0.105** | 0.135** | 0.140** | 0.360** | 1.000 | 0.186** |
| Alcohol beverages | -0.015 | -0.014 | -0.002 | 0.002 | 0.038 | 0.121** | 0.106** | 0.126** | 0.190** | 0.186** | 1.000 |
| Note: Data weighted by countries.**. Correlation is significant at the 0.01 level (2-tailed). *. Correlation is significant at the 0.05 level (2-tailed). | | | | | | | | | | | |

Supplementary Table 2. Pairwise comparison analysis of estimated marginal means between household groups within each food consumption category COVID 19 affected change.

| Variable | | | Mean Difference (I-J) | Std. Error | Sig.^b^ | 95% Confidence Interval for Difference^b^ | |
| --- | --- | --- | --- | --- | --- | --- | --- |
|  |  |  |  |  |  | Lower Bound | Upper Bound |
| Fruit and vegetables | Households with children 0-19 | Single-person households | -0.069^*^ | 0.024 | 0.013 | -0.127 | -0.011 |
|  |  | Households with two or more adults without children | -0.045 | 0.021 | 0.095 | -0.096 | 0.005 |
|  | Single-person households | Households with children 0-19 | 0.069^*^ | 0.024 | 0.013 | 0.011 | 0.127 |
|  |  | Households with two or more adults without children | 0.023 | 0.022 | 0.647 | -0.030 | 0.077 |
|  | Households with two or more adults without children | Households with children 0-19 | 0.045 | 0.021 | 0.095 | -0.005 | 0.096 |
|  |  | Single-person households | -0.023 | 0.022 | 0.647 | -0.077 | 0.030 |
| Meat and meat products | Households with children 0-19 | Single-person households | -0.067^*^ | 0.024 | 0.016 | -0.124 | -0.010 |
|  |  | Households with two or more adults without children | -0.058^*^ | 0.021 | 0.020 | -0.108 | -0.007 |
|  | Single-person households | Households with children 0-19 | 0.067^*^ | 0.024 | 0.016 | 0.010 | 0.124 |
|  |  | Households with two or more adults without children | 0.009 | 0.022 | 0.965 | -0.044 | 0.062 |
|  | Households with two or more adults without children | Households with children 0-19 | 0.058^*^ | 0.021 | 0.020 | 0.007 | 0.108 |
|  |  | Single-person households | -0.009 | 0.022 | 0.965 | -0.062 | 0.044 |
| Fish and fish products | Households with children 0-19 | Single-person households | -0.021 | 0.027 | 0.828 | -0.085 | 0.044 |
|  |  | Households with two or more adults without children | -0.040 | 0.024 | 0.257 | -0.097 | 0.017 |
|  | Single-person households | Households with children 0-19 | 0.021 | 0.027 | 0.828 | -0.044 | 0.085 |
|  |  | Households with two or more adults without children | -0.019 | 0.025 | 0.827 | -0.079 | 0.041 |
|  | Households with two or more adults without children | Households with children 0-19 | 0.040 | 0.024 | 0.257 | -0.017 | 0.097 |
|  |  | Single-person households | 0.019 | 0.025 | 0.827 | -0.041 | 0.079 |
| Bread and bakery products | Households with children 0-19 | Single-person households | -0.073^*^ | 0.030 | 0.042 | -0.144 | -0.002 |
|  |  | Households with two or more adults without children | -0.146^*^ | 0.026 | 0.000 | -0.209 | -0.084 |
|  | Single-person households | Households with children 0-19 | 0.073^*^ | 0.030 | 0.042 | 0.002 | 0.144 |
|  |  | Households with two or more adults without children | -0.073^*^ | 0.027 | 0.023 | -0.139 | -0.008 |
|  | Households with two or more adults without children | Households with children 0-19 | 0.146^*^ | 0.026 | 0.000 | 0.084 | 0.209 |
|  |  | Single-person households | 0.073^*^ | 0.027 | 0.023 | 0.008 | 0.139 |
| Dairy products | Households with children 0-19 | Single-person households | -0.054 | 0.025 | 0.089 | -0.114 | 0.006 |
|  |  | Households with two or more adults without children | -0.059^*^ | 0.022 | 0.022 | -0.111 | -0.006 |
|  | Single-person households | Households with children 0-19 | 0.054 | 0.025 | 0.089 | -0.006 | 0.114 |
|  |  | Households with two or more adults without children | -0.005 | 0.023 | 0.995 | -0.060 | 0.050 |
|  | Households with two or more adults without children | Households with children 0-19 | 0.059^*^ | 0.022 | 0.022 | 0.006 | 0.111 |
|  |  | Single-person households | 0.005 | 0.023 | 0.995 | -0.050 | 0.060 |
| Frozen food | Households with children 0-19 | Single-person households | -0.056 | 0.031 | 0.197 | -0.130 | 0.018 |
|  |  | Households with two or more adults without children | 0.045 | 0.027 | 0.262 | -0.020 | 0.111 |
|  | Single-person households | Households with children 0-19 | 0.056 | 0.031 | 0.197 | -0.018 | 0.130 |
|  |  | Households with two or more adults without children | 0.102^*^ | 0.029 | 0.001 | 0.033 | 0.170 |
|  | Households with two or more adults without children | Households with children 0-19 | -0.045 | 0.027 | 0.262 | -0.111 | 0.020 |
|  |  | Single-person households | -0.102^*^ | 0.029 | 0.001 | -0.170 | -0.033 |
| Canned food | Households with children 0-19 | Single-person households | -0.063 | 0.028 | 0.081 | -0.131 | 0.005 |
|  |  | Households with two or more adults without children | -0.035 | 0.025 | 0.423 | -0.095 | 0.025 |
|  | Single-person households | Households with children 0-19 | 0.063 | 0.028 | 0.081 | -0.005 | 0.131 |
|  |  | Households with two or more adults without children | 0.028 | 0.026 | 0.638 | -0.035 | 0.091 |
|  | Households with two or more adults without children | Households with children 0-19 | 0.035 | 0.025 | 0.423 | -0.025 | 0.095 |
|  |  | Single-person households | -0.028 | 0.026 | 0.638 | -0.091 | 0.035 |
| Ready Made Meals | Households with children 0-19 | Single-person households | -0.013 | 0.033 | 0.971 | -0.091 | 0.065 |
|  |  | Households with two or more adults without children | -0.042 | 0.029 | 0.372 | -0.111 | 0.027 |
|  | Single-person households | Households with children 0-19 | 0.013 | 0.033 | 0.971 | -0.065 | 0.091 |
|  |  | Households with two or more adults without children | -0.029 | 0.030 | 0.704 | -0.102 | 0.043 |
|  | Households with two or more adults without children | Households with children 0-19 | 0.042 | 0.029 | 0.372 | -0.027 | 0.111 |
|  |  | Single-person households | 0.029 | 0.030 | 0.704 | -0.043 | 0.102 |
| Cake and Biscuits | Households with children 0-19 | Single-person households | 0.110^*^ | 0.034 | 0.003 | 0.030 | 0.190 |
|  |  | Households with two or more adults without children | 0.034 | 0.030 | 0.572 | -0.036 | 0.105 |
|  | Single-person households | Households with children 0-19 | -0.110^*^ | 0.034 | 0.003 | -0.190 | -0.030 |
|  |  | Households with two or more adults without children | -0.076^*^ | 0.031 | 0.043 | -0.150 | -0.002 |
|  | Households with two or more adults without children | Households with children 0-19 | -0.034 | 0.030 | 0.572 | -0.105 | 0.036 |
|  |  | Single-person households | 0.076^*^ | 0.031 | 0.043 | 0.002 | 0.150 |
| Sweets | Households with children 0-19 | Single-person households | 0.132^*^ | 0.033 | 0.000 | 0.053 | 0.211 |
|  |  | Households with two or more adults without children | 0.082^*^ | 0.029 | 0.016 | 0.012 | 0.152 |
|  | Single-person households | Households with children 0-19 | -0.132^*^ | 0.033 | 0.000 | -0.211 | -0.053 |
|  |  | Households with two or more adults without children | -0.050 | 0.031 | 0.275 | -0.123 | 0.023 |
|  | Households with two or more adults without children | Households with children 0-19 | -0.082^*^ | 0.029 | 0.016 | -0.152 | -0.012 |
|  |  | Single-person households | 0.050 | 0.031 | 0.275 | -0.023 | 0.123 |
| Alcohol beverages | Households with children 0-19 | Single-person households | 0.033 | 0.029 | 0.576 | -0.035 | 0.101 |
|  |  | Households with two or more adults without children | -0.015 | 0.025 | 0.907 | -0.075 | 0.045 |
|  | Single-person households | Households with children 0-19 | -0.033 | 0.029 | 0.576 | -0.101 | 0.035 |
|  |  | Households with two or more adults without children | -0.048 | 0.026 | 0.191 | -0.111 | 0.015 |
|  | Households with two or more adults without children | Households with children 0-19 | 0.015 | 0.025 | 0.907 | -0.045 | 0.075 |
|  |  | Single-person households | 0.048 | 0.026 | 0.191 | -0.015 | 0.111 |
| Estimated marginal means using MANOVA procedure, data weighted by countries. | | | | | | | |
| *. The mean difference is significant at the .05 level. | | | | | | | |
| b. Adjustment for multiple comparisons: Sidak. | | | | | | | |

Supplementary Table 3. Pairwise comparison analysis of estimated marginal means between household groups within each food purchasing category COVID 19 affected changes.

| Variable | | | Mean Difference (I-J) | Std. Error | Sig.^b^ | 95% Confidence Interval for Difference^b^ | |
| --- | --- | --- | --- | --- | --- | --- | --- |
|  |  |  |  |  |  | Lower Bound | Upper Bound |
| Fruits and vegetables | Households with children 0-19 | Single-person households | -0.137^*^ | 0.027 | 0.000 | -0.203 | -0.072 |
|  |  | Households with two or more adults without children | -0.039 | 0.024 | 0.281 | -0.097 | 0.018 |
|  | Single-person households | Households with children 0-19 | 0.137^*^ | 0.027 | 0.000 | 0.072 | 0.203 |
|  |  | Households with two or more adults without children | 0.098^*^ | 0.025 | 0.000 | 0.038 | 0.159 |
|  | Households with two or more adults without children | Households with children 0-19 | 0.039 | 0.024 | 0.281 | -0.018 | 0.097 |
|  |  | Single-person households | -0.098^*^ | 0.025 | 0.000 | -0.159 | -0.038 |
| Meat and meat products | Households with children 0-19 | Single-person households | -0.127^*^ | 0.026 | 0.000 | -0.188 | -0.065 |
|  |  | Households with two or more adults without children | -0.020 | 0.023 | 0.750 | -0.074 | 0.034 |
|  | Single-person households | Households with children 0-19 | 0.127^*^ | 0.026 | 0.000 | 0.065 | 0.188 |
|  |  | Households with two or more adults without children | 0.106^*^ | 0.024 | 0.000 | 0.050 | 0.163 |
|  | Households with two or more adults without children | Households with children 0-19 | 0.020 | 0.023 | 0.750 | -0.034 | 0.074 |
|  |  | Single-person households | -0.106^*^ | 0.024 | 0.000 | -0.163 | -0.050 |
| Other fresh food products | Households with children 0-19 | Single-person households | -0.135^*^ | 0.029 | 0.000 | -0.204 | -0.066 |
|  |  | Households with two or more adults without children | -0.056 | 0.025 | 0.082 | -0.116 | 0.005 |
|  | Single-person households | Households with children 0-19 | 0.135^*^ | 0.029 | 0.000 | 0.066 | 0.204 |
|  |  | Households with two or more adults without children | 0.079^*^ | 0.027 | 0.009 | 0.015 | 0.143 |
|  | Households with two or more adults without children | Households with children 0-19 | 0.056 | 0.025 | 0.082 | -0.005 | 0.116 |
|  |  | Single-person households | -0.079^*^ | 0.027 | 0.009 | -0.143 | -0.015 |
| Other non fresh food products | Households with children 0-19 | Single-person households | -0.074^*^ | 0.025 | 0.010 | -0.134 | -0.014 |
|  |  | Households with two or more adults without children | -0.046 | 0.022 | 0.104 | -0.099 | 0.006 |
|  | Single-person households | Households with children 0-19 | 0.074^*^ | 0.025 | 0.010 | 0.014 | 0.134 |
|  |  | Households with two or more adults without children | 0.027 | 0.023 | 0.561 | -0.028 | 0.083 |
|  | Households with two or more adults without children | Households with children 0-19 | 0.046 | 0.022 | 0.104 | -0.006 | 0.099 |
|  |  | Single-person households | -0.027 | 0.023 | 0.561 | -0.083 | 0.028 |
| Estimated marginal means using MANOVA procedure, data weighted by countries | | | | | | | |
| *. The mean difference is significant at the .05 level. | | | | | | | |
| b. Adjustment for multiple comparisons: Sidak. | | | | | | | |

Supplementary Table 4. Pairwise comparison analysis of estimated marginal means between AIC groups within each food consumption category COVID 19 affected changes.

| Variable | | | Mean Difference (I-J) | Std. Error | Sig.^b^ | 95% Confidence Interval for Difference^b^ | |
| --- | --- | --- | --- | --- | --- | --- | --- |
|  |  |  |  |  |  | Lower Bound | Upper Bound |
| Fruit and vegetables | Very low | Low | 0.056 | 0.054 | 0.883 | -0.086 | 0.198 |
|  |  | High | 0.122 | 0.051 | 0.099 | -0.013 | 0.256 |
|  |  | Very high | 0.079 | 0.051 | 0.553 | -0.057 | 0.214 |
|  | Low | Very low | -0.056 | 0.054 | 0.883 | -0.198 | 0.086 |
|  |  | High | 0.066 | 0.026 | 0.062 | -0.002 | 0.134 |
|  |  | Very high | 0.023 | 0.026 | 0.946 | -0.047 | 0.093 |
|  | High | Very low | -0.122 | 0.051 | 0.099 | -0.256 | 0.013 |
|  |  | Low | -0.066 | 0.026 | 0.062 | -0.134 | 0.002 |
|  |  | Very high | -0.043 | 0.020 | 0.175 | -0.095 | 0.010 |
|  | Very high | Very low | -0.079 | 0.051 | 0.553 | -0.214 | 0.057 |
|  |  | Low | -0.023 | 0.026 | 0.946 | -0.093 | 0.047 |
|  |  | High | 0.043 | 0.020 | 0.175 | -0.010 | 0.095 |
| Meat and meat products | Very low | Low | -0.034 | 0.054 | 0.989 | -0.175 | 0.107 |
|  |  | High | 0.042 | 0.051 | 0.957 | -0.092 | 0.176 |
|  |  | Very high | 0.023 | 0.051 | 0.998 | -0.112 | 0.158 |
|  | Low | Very low | 0.034 | 0.054 | 0.989 | -0.107 | 0.175 |
|  |  | High | 0.076^*^ | 0.026 | 0.018 | 0.008 | 0.144 |
|  |  | Very high | 0.057 | 0.026 | 0.173 | -0.013 | 0.126 |
|  | High | Very low | -0.042 | 0.051 | 0.957 | -0.176 | 0.092 |
|  |  | Low | -0.076^*^ | 0.026 | 0.018 | -0.144 | -0.008 |
|  |  | Very high | -0.019 | 0.020 | 0.910 | -0.072 | 0.033 |
|  | Very high | Very low | -0.023 | 0.051 | 0.998 | -0.158 | 0.112 |
|  |  | Low | -0.057 | 0.026 | 0.173 | -0.126 | 0.013 |
|  |  | High | 0.019 | 0.020 | 0.910 | -0.033 | 0.072 |
| Fish and fish products | Very low | Low | 0.120 | 0.060 | 0.253 | -0.039 | 0.279 |
|  |  | High | -0.014 | 0.057 | 1.000 | -0.165 | 0.137 |
|  |  | Very high | -0.130 | 0.058 | 0.136 | -0.282 | 0.022 |
|  | Low | Very low | -0.120 | 0.060 | 0.253 | -0.279 | 0.039 |
|  |  | High | -0.134^*^ | 0.029 | 0.000 | -0.210 | -0.058 |
|  |  | Very high | -0.250^*^ | 0.030 | 0.000 | -0.328 | -0.172 |
|  | High | Very low | 0.014 | 0.057 | 1.000 | -0.137 | 0.165 |
|  |  | Low | 0.134^*^ | 0.029 | 0.000 | 0.058 | 0.210 |
|  |  | Very high | -0.116^*^ | 0.022 | 0.000 | -0.175 | -0.057 |
|  | Very high | Very low | 0.130 | 0.058 | 0.136 | -0.022 | 0.282 |
|  |  | Low | 0.250^*^ | 0.030 | 0.000 | 0.172 | 0.328 |
|  |  | High | 0.116^*^ | 0.022 | 0.000 | 0.057 | 0.175 |
| Bread and bakery products | Very low | Low | 0.124 | 0.067 | 0.317 | -0.051 | 0.300 |
|  |  | High | 0.078 | 0.063 | 0.771 | -0.088 | 0.243 |
|  |  | Very high | 0.024 | 0.063 | 0.999 | -0.143 | 0.191 |
|  | Low | Very low | -0.124 | 0.067 | 0.317 | -0.300 | 0.051 |
|  |  | High | -0.047 | 0.032 | 0.600 | -0.130 | 0.037 |
|  |  | Very high | -0.100^*^ | 0.033 | 0.012 | -0.186 | -0.015 |
|  | High | Very low | -0.078 | 0.063 | 0.771 | -0.243 | 0.088 |
|  |  | Low | 0.047 | 0.032 | 0.600 | -0.037 | 0.130 |
|  |  | Very high | -0.054 | 0.025 | 0.162 | -0.118 | 0.011 |
|  | Very high | Very low | -0.024 | 0.063 | 0.999 | -0.191 | 0.143 |
|  |  | Low | 0.100^*^ | 0.033 | 0.012 | 0.015 | 0.186 |
|  |  | High | 0.054 | 0.025 | 0.162 | -0.011 | 0.118 |
| Dairy products | Very low | Low | -0.077 | 0.056 | 0.668 | -0.224 | 0.070 |
|  |  | High | -0.112 | 0.053 | 0.188 | -0.251 | 0.027 |
|  |  | Very high | -0.100 | 0.053 | 0.316 | -0.240 | 0.040 |
|  | Low | Very low | 0.077 | 0.056 | 0.668 | -0.070 | 0.224 |
|  |  | High | -0.035 | 0.027 | 0.716 | -0.105 | 0.035 |
|  |  | Very high | -0.023 | 0.027 | 0.957 | -0.095 | 0.049 |
|  | High | Very low | 0.112 | 0.053 | 0.188 | -0.027 | 0.251 |
|  |  | Low | 0.035 | 0.027 | 0.716 | -0.035 | 0.105 |
|  |  | Very high | 0.012 | 0.021 | 0.991 | -0.042 | 0.067 |
|  | Very high | Very low | 0.100 | 0.053 | 0.316 | -0.040 | 0.240 |
|  |  | Low | 0.023 | 0.027 | 0.957 | -0.049 | 0.095 |
|  |  | High | -0.012 | 0.021 | 0.991 | -0.067 | 0.042 |
| Frozen Food | Very low | Low | -0.106 | 0.070 | 0.558 | -0.289 | 0.077 |
|  |  | High | -0.110 | 0.066 | 0.453 | -0.283 | 0.064 |
|  |  | Very high | -0.019 | 0.066 | 1.000 | -0.193 | 0.155 |
|  | Low | Very low | 0.106 | 0.070 | 0.558 | -0.077 | 0.289 |
|  |  | High | -0.004 | 0.033 | 1.000 | -0.091 | 0.084 |
|  |  | Very high | 0.087 | 0.034 | 0.063 | -0.003 | 0.177 |
|  | High | Very low | 0.110 | 0.066 | 0.453 | -0.064 | 0.283 |
|  |  | Low | 0.004 | 0.033 | 1.000 | -0.084 | 0.091 |
|  |  | Very high | 0.091^*^ | 0.026 | 0.003 | 0.023 | 0.158 |
|  | Very high | Very low | 0.019 | 0.066 | 1.000 | -0.155 | 0.193 |
|  |  | Low | -0.087 | 0.034 | 0.063 | -0.177 | 0.003 |
|  |  | High | -0.091^*^ | 0.026 | 0.003 | -0.158 | -0.023 |
| Canned Food | Very low | Low | -0.168 | 0.064 | 0.051 | -0.335 | 0.000 |
|  |  | High | -0.249^*^ | 0.060 | 0.000 | -0.408 | -0.090 |
|  |  | Very high | -0.167^*^ | 0.061 | 0.035 | -0.327 | -0.007 |
|  | Low | Very low | 0.168 | 0.064 | 0.051 | 0.000 | 0.335 |
|  |  | High | -0.082^*^ | 0.030 | 0.043 | -0.162 | -0.002 |
|  |  | Very high | 0.000 | 0.031 | 1.000 | -0.082 | 0.083 |
|  | High | Very low | 0.249^*^ | 0.060 | 0.000 | 0.090 | 0.408 |
|  |  | Low | 0.082^*^ | 0.030 | 0.043 | 0.002 | 0.162 |
|  |  | Very high | 0.082^*^ | 0.024 | 0.003 | 0.020 | 0.144 |
|  | Very high | Very low | 0.167^*^ | 0.061 | 0.035 | 0.007 | 0.327 |
|  |  | Low | 0.000 | 0.031 | 1.000 | -0.083 | 0.082 |
|  |  | High | -0.082^*^ | 0.024 | 0.003 | -0.144 | -0.020 |
| Ready Made Meals | Very low | Low | -0.027 | 0.072 | 0.999 | -0.217 | 0.163 |
|  |  | High | -0.043 | 0.068 | 0.989 | -0.223 | 0.137 |
|  |  | Very high | -0.286^*^ | 0.069 | 0.000 | -0.468 | -0.105 |
|  | Low | Very low | 0.027 | 0.072 | 0.999 | -0.163 | 0.217 |
|  |  | High | -0.016 | 0.035 | 0.998 | -0.107 | 0.075 |
|  |  | Very high | -0.259^*^ | 0.035 | 0.000 | -0.352 | -0.166 |
|  | High | Very low | 0.043 | 0.068 | 0.989 | -0.137 | 0.223 |
|  |  | Low | 0.016 | 0.035 | 0.998 | -0.075 | 0.107 |
|  |  | Very high | -0.243^*^ | 0.027 | 0.000 | -0.313 | -0.173 |
|  | Very high | Very low | 0.286^*^ | 0.069 | 0.000 | 0.105 | 0.468 |
|  |  | Low | 0.259^*^ | 0.035 | 0.000 | 0.166 | 0.352 |
|  |  | High | 0.243^*^ | 0.027 | 0.000 | 0.173 | 0.313 |
| Cake Biscuits | Very low | Low | -0.313^*^ | 0.074 | 0.000 | -0.509 | -0.117 |
|  |  | High | -0.221^*^ | 0.071 | 0.011 | -0.406 | -0.035 |
|  |  | Very high | -0.133 | 0.071 | 0.313 | -0.320 | 0.054 |
|  | Low | Very low | 0.313^*^ | 0.074 | 0.000 | 0.117 | 0.509 |
|  |  | High | 0.092 | 0.036 | 0.058 | -0.002 | 0.186 |
|  |  | Very high | 0.179^*^ | 0.036 | 0.000 | 0.083 | 0.275 |
|  | High | Very low | 0.221^*^ | 0.071 | 0.011 | 0.035 | 0.406 |
|  |  | Low | -0.092 | 0.036 | 0.058 | -0.186 | 0.002 |
|  |  | Very high | 0.087^*^ | 0.028 | 0.009 | 0.015 | 0.160 |
|  | Very high | Very low | 0.133 | 0.071 | 0.313 | -0.054 | 0.320 |
|  |  | Low | -0.179^*^ | 0.036 | 0.000 | -0.275 | -0.083 |
|  |  | High | -0.087^*^ | 0.028 | 0.009 | -0.160 | -0.015 |
| Sweets | Very low | Low | -0.349^*^ | 0.074 | 0.000 | -0.544 | -0.154 |
|  |  | High | -0.364^*^ | 0.070 | 0.000 | -0.548 | -0.179 |
|  |  | Very high | -0.258^*^ | 0.071 | 0.002 | -0.444 | -0.073 |
|  | Low | Very low | 0.349^*^ | 0.074 | 0.000 | 0.154 | 0.544 |
|  |  | High | -0.015 | 0.035 | 0.999 | -0.108 | 0.078 |
|  |  | Very high | 0.090 | 0.036 | 0.075 | -0.005 | 0.186 |
|  | High | Very low | 0.364^*^ | 0.070 | 0.000 | 0.179 | 0.548 |
|  |  | Low | 0.015 | 0.035 | 0.999 | -0.078 | 0.108 |
|  |  | Very high | 0.105^*^ | 0.027 | 0.001 | 0.033 | 0.177 |
|  | Very high | Very low | 0.258^*^ | 0.071 | 0.002 | 0.073 | 0.444 |
|  |  | Low | -0.090 | 0.036 | 0.075 | -0.186 | 0.005 |
|  |  | High | -0.105^*^ | 0.027 | 0.001 | -0.177 | -0.033 |
| Alcohol beverages | Very low | Low | -0.151 | 0.064 | 0.101 | -0.318 | 0.016 |
|  |  | High | -0.252^*^ | 0.060 | 0.000 | -0.410 | -0.093 |
|  |  | Very high | -0.177^*^ | 0.061 | 0.021 | -0.336 | -0.017 |
|  | Low | Very low | 0.151 | 0.064 | 0.101 | -0.016 | 0.318 |
|  |  | High | -0.101^*^ | 0.030 | 0.006 | -0.180 | -0.021 |
|  |  | Very high | -0.026 | 0.031 | 0.959 | -0.107 | 0.056 |
|  | High | Very low | 0.252^*^ | 0.060 | 0.000 | 0.093 | 0.410 |
|  |  | Low | 0.101^*^ | 0.030 | 0.006 | 0.021 | 0.180 |
|  |  | Very high | 0.075^*^ | 0.023 | 0.008 | 0.013 | 0.137 |
|  | Very high | Very low | 0.177^*^ | 0.061 | 0.021 | 0.017 | 0.336 |
|  |  | Low | 0.026 | 0.031 | 0.959 | -0.056 | 0.107 |
|  |  | High | -0.075^*^ | 0.023 | 0.008 | -0.137 | -0.013 |
| Estimated marginal means using MANOVA procedure, data weighted by countries. | | | | | | | |
| *. The mean difference is significant at the .05 level. | | | | | | | |
| b. Adjustment for multiple comparisons: Sidak. | | | | | | | |

Supplementary Table 5. Pairwise comparison analysis of estimated marginal means between AIC groups within each food purchasing category COVID 19 affected changes.

| Variable | | | Mean Difference (I-J) | Std. Error | Sig.^b^ | 95% Confidence Interval for Difference^b^ | |
| --- | --- | --- | --- | --- | --- | --- | --- |
|  |  |  |  |  |  | Lower Bound | Upper Bound |
| Fruits and vegetables | Very low | Low | -0.033 | 0.061 | 0.995 | -0.193 | 0.126 |
|  |  | High | -0.148 | 0.057 | 0.058 | -0.299 | 0.003 |
|  |  | Very high | -0.373^*^ | 0.058 | 0.000 | -0.525 | -0.221 |
|  | Low | Very low | 0.033 | 0.061 | 0.995 | -0.126 | 0.193 |
|  |  | High | -0.115^*^ | 0.029 | 0.000 | -0.191 | -0.038 |
|  |  | Very high | -0.339^*^ | 0.030 | 0.000 | -0.418 | -0.261 |
|  | High | Very low | 0.148 | 0.057 | 0.058 | -0.003 | 0.299 |
|  |  | Low | 0.115^*^ | 0.029 | 0.000 | 0.038 | 0.191 |
|  |  | Very high | -0.225^*^ | 0.022 | 0.000 | -0.284 | -0.166 |
|  | Very high | Very low | 0.373^*^ | 0.058 | 0.000 | 0.221 | 0.525 |
|  |  | Low | 0.339^*^ | 0.030 | 0.000 | 0.261 | 0.418 |
|  |  | High | 0.225^*^ | 0.022 | 0.000 | 0.166 | 0.284 |
| Meat and meat products | Very low | Low | -0.081 | 0.057 | 0.634 | -0.230 | 0.068 |
|  |  | High | -0.094 | 0.054 | 0.389 | -0.236 | 0.047 |
|  |  | Very high | -0.313^*^ | 0.054 | 0.000 | -0.456 | -0.171 |
|  | Low | Very low | 0.081 | 0.057 | 0.634 | -0.068 | 0.230 |
|  |  | High | -0.014 | 0.027 | 0.997 | -0.085 | 0.058 |
|  |  | Very high | -0.232^*^ | 0.028 | 0.000 | -0.306 | -0.159 |
|  | High | Very low | 0.094 | 0.054 | 0.389 | -0.047 | 0.236 |
|  |  | Low | 0.014 | 0.027 | 0.997 | -0.058 | 0.085 |
|  |  | Very high | -0.219^*^ | 0.021 | 0.000 | -0.274 | -0.164 |
|  | Very high | Very low | 0.313^*^ | 0.054 | 0.000 | 0.171 | 0.456 |
|  |  | Low | 0.232^*^ | 0.028 | 0.000 | 0.159 | 0.306 |
|  |  | High | 0.219^*^ | 0.021 | 0.000 | 0.164 | 0.274 |
| Other fresh food products | Very low | Low | -0.043 | 0.063 | 0.982 | -0.209 | 0.122 |
|  |  | High | -0.292^*^ | 0.060 | 0.000 | -0.449 | -0.135 |
|  |  | Very high | -0.554^*^ | 0.060 | 0.000 | -0.712 | -0.396 |
|  | Low | Very low | 0.043 | 0.063 | 0.982 | -0.122 | 0.209 |
|  |  | High | -0.249^*^ | 0.030 | 0.000 | -0.328 | -0.170 |
|  |  | Very high | -0.511^*^ | 0.031 | 0.000 | -0.592 | -0.429 |
|  | High | Very low | 0.292^*^ | 0.060 | 0.000 | 0.135 | 0.449 |
|  |  | Low | 0.249^*^ | 0.030 | 0.000 | 0.170 | 0.328 |
|  |  | Very high | -0.262^*^ | 0.023 | 0.000 | -0.323 | -0.201 |
|  | Very high | Very low | 0.554^*^ | 0.060 | 0.000 | 0.396 | 0.712 |
|  |  | Low | 0.511^*^ | 0.031 | 0.000 | 0.429 | 0.592 |
|  |  | High | 0.262^*^ | 0.023 | 0.000 | 0.201 | 0.323 |
| Other non fresh food products | Very low | Low | -0.014 | 0.056 | 1.000 | -0.162 | 0.133 |
|  |  | High | -0.017 | 0.053 | 1.000 | -0.157 | 0.122 |
|  |  | Very high | -0.132 | 0.053 | 0.079 | -0.272 | 0.009 |
|  | Low | Very low | 0.014 | 0.056 | 1.000 | -0.133 | 0.162 |
|  |  | High | -0.003 | 0.027 | 1.000 | -0.074 | 0.067 |
|  |  | Very high | -0.118^*^ | 0.027 | 0.000 | -0.190 | -0.045 |
|  | High | Very low | 0.017 | 0.053 | 1.000 | -0.122 | 0.157 |
|  |  | Low | 0.003 | 0.027 | 1.000 | -0.067 | 0.074 |
|  |  | Very high | -0.114^*^ | 0.021 | 0.000 | -0.169 | -0.060 |
|  | Very high | Very low | 0.132 | 0.053 | 0.079 | -0.009 | 0.272 |
|  |  | Low | 0.118^*^ | 0.027 | 0.000 | 0.045 | 0.190 |
|  |  | High | 0.114^*^ | 0.021 | 0.000 | 0.060 | 0.169 |
| Estimated marginal means using MANOVA procedure, data weighted by countries. | | | | | | | |
| *. The mean difference is significant at the .05 level. | | | | | | | |
| b. Adjustment for multiple comparisons: Sidak. | | | | | | | |

Supplementary Table 6. Estimated marginal means of interaction between AIC groups for each food purchasing category COVID 19 affected change.

| Dependent Variable | AIC | Household composition | Mean | Std. Error | 95% Confidence Interval | |
| --- | --- | --- | --- | --- | --- | --- |
|  |  |  |  |  | Lower Bound | Upper Bound |
| Fruits and vegetables | Very low | Households with children 0-19 | -0.750 | 0.095 | -0.937 | -0.563 |
|  |  | Single-person households | -0.263 | 0.131 | -0.520 | -0.006 |
|  |  | Households with two or more adults without children | -0.740 | 0.081 | -0.898 | -0.582 |
|  | Low | Households with children 0-19 | -0.603 | 0.039 | -0.678 | -0.527 |
|  |  | Single-person households | -0.590 | 0.056 | -0.700 | -0.481 |
|  |  | Households with two or more adults without children | -0.688 | 0.042 | -0.769 | -0.606 |
|  | High | Households with children 0-19 | -0.533 | 0.028 | -0.587 | -0.479 |
|  |  | Single-person households | -0.451 | 0.031 | -0.512 | -0.390 |
|  |  | Households with two or more adults without children | -0.513 | 0.023 | -0.558 | -0.469 |
|  | Very high | Households with children 0-19 | -0.345 | 0.034 | -0.411 | -0.279 |
|  |  | Single-person households | -0.222 | 0.030 | -0.281 | -0.163 |
|  |  | Households with two or more adults without children | -0.296 | 0.025 | -0.344 | -0.248 |
| Meat and meat products | Very low | Households with children 0-19 | -0.833 | 0.089 | -1.009 | -0.658 |
|  |  | Single-person households | -0.105 | 0.123 | -0.346 | 0.136 |
|  |  | Households with two or more adults without children | -0.400 | 0.076 | -0.549 | -0.251 |
|  | Low | Households with children 0-19 | -0.393 | 0.036 | -0.464 | -0.322 |
|  |  | Single-person households | -0.410 | 0.052 | -0.512 | -0.307 |
|  |  | Households with two or more adults without children | -0.455 | 0.039 | -0.532 | -0.379 |
|  | High | Households with children 0-19 | -0.426 | 0.026 | -0.477 | -0.375 |
|  |  | Single-person households | -0.293 | 0.029 | -0.350 | -0.236 |
|  |  | Households with two or more adults without children | -0.439 | 0.021 | -0.481 | -0.397 |
|  | Very high | Households with children 0-19 | -0.203 | 0.032 | -0.265 | -0.141 |
|  |  | Single-person households | -0.152 | 0.028 | -0.207 | -0.096 |
|  |  | Households with two or more adults without children | -0.197 | 0.023 | -0.242 | -0.152 |
| Other fresh food products | Very low | Households with children 0-19 | -0.806 | 0.099 | -1.000 | -0.611 |
|  |  | Single-person households | -0.684 | 0.136 | -0.951 | -0.417 |
|  |  | Households with two or more adults without children | -0.840 | 0.084 | -1.005 | -0.675 |
|  | Low | Households with children 0-19 | -0.753 | 0.040 | -0.832 | -0.675 |
|  |  | Single-person households | -0.724 | 0.058 | -0.837 | -0.610 |
|  |  | Households with two or more adults without children | -0.788 | 0.043 | -0.873 | -0.704 |
|  | High | Households with children 0-19 | -0.488 | 0.029 | -0.545 | -0.432 |
|  |  | Single-person households | -0.492 | 0.032 | -0.555 | -0.429 |
|  |  | Households with two or more adults without children | -0.520 | 0.024 | -0.566 | -0.474 |
|  | Very high | Households with children 0-19 | -0.342 | 0.035 | -0.410 | -0.273 |
|  |  | Single-person households | -0.164 | 0.031 | -0.225 | -0.103 |
|  |  | Households with two or more adults without children | -0.240 | 0.026 | -0.291 | -0.190 |
| Other non fresh food products | Very low | Households with children 0-19 | -0.194 | 0.088 | -0.367 | -0.022 |
|  |  | Single-person households | -0.105 | 0.121 | -0.343 | 0.133 |
|  |  | Households with two or more adults without children | -0.300 | 0.075 | -0.447 | -0.153 |
|  | Low | Households with children 0-19 | -0.224 | 0.036 | -0.294 | -0.154 |
|  |  | Single-person households | -0.181 | 0.052 | -0.282 | -0.080 |
|  |  | Households with two or more adults without children | -0.228 | 0.038 | -0.303 | -0.152 |
|  | High | Households with children 0-19 | -0.249 | 0.026 | -0.300 | -0.199 |
|  |  | Single-person households | -0.204 | 0.029 | -0.260 | -0.147 |
|  |  | Households with two or more adults without children | -0.214 | 0.021 | -0.255 | -0.173 |
|  | Very high | Households with children 0-19 | -0.161 | 0.031 | -0.223 | -0.100 |
|  |  | Single-person households | -0.074 | 0.028 | -0.129 | -0.020 |
|  |  | Households with two or more adults without children | -0.083 | 0.023 | -0.127 | -0.038 |

Note: Estimated marginal means using MANOVA procedure, data weighted by countries.
