# Supplementary material for: European household spending and socio-economic impacts on food behavior during the first wave of COVID-19
Source: Front Nutr. 2022 Aug 3;9:869091. doi: 10.3389/fnut.2022.869091 (PMC9382126; doi:10.3389/fnut.2022.869091)
Supplement: Supplementary file 2 [file Data_Sheet_2.pdf]

Seite 01
intro

V189

OUR RELATIONSHIP WITH FOOD DURING THE COVID-19 PANDEMIC

This short European-wide survey is designed to capture important aspects of your behavior and attitudes to food during this time of pandemic. Your participation will be highly appreciated and help us to understand how food systems, as a central aspect of our lives, can be improved for everyone's benefit over the longer term.

The questionnaire takes about **10-15 minutes** to complete.

Unless where clearly stated otherwise, please answer on behalf of all the people in or connected to your household who are typically involved together in obtaining, preparing and eating food, not just yourself as an individual. If there is someone else in your household who has more knowledge than you about some of the questions, please feel free to ask her or him. Please ensure that only one response is submitted per household.

Please note:

- The results of this research will be used for scientific purposes only and may be published.
- All data will be treated anonymously and confidentially at every stage of the research.
- Only general statistics will appear in reports.
- This questionnaire is 100% compliant with the EU's GDPR regulations. Please click here for more information.
- As an independent research initiative, it is entirely free from any political, commercial or vested interest.
- You have the right to discontinue the survey at any time.

V101

- ○ **I understand and accept the points stated above and wish to participate in this study.**

Seite 02
fg

Seite 03
screen

MQ44

- ○ yes
- ○ no

Seite 04

Seite 05
start

V133

In the following questions, we are interested in your behaviour both before and during the Covid-19 pandemic.

By the term during Covid-19, we mean the situation right now.

Seite 06
obtain

MQ28

1. What are the main ways your household´s food is obtained before and during Covid-19?

Click as many options as are relevant!

Before Covid-19 (V102)	During Covid-19 (V103)
☐ Own purchase	☐ Own purchase
☐ Food banks, charities	☐ Food banks, charities
☐ Grow own	☐ Grow own
☐ Other (please specify):	☐ Other (please specify):

Seite 07
Source

MQ29

2. What are the main sources of buying your household´s FRESH VEGETABLES / FRUITS before and during Covid-19?

Click as many options as are relevant!

Before Covid-19 (MQ20)	During Covid-19 (MQ21)
☐ Big supermarkets	☐ Big supermarkets
☐ Supermarkets	☐ Supermarkets
☐ Discount shops	☐ Discount shops
☐ Bakeries, butchers, small grocery shops	☐ Bakeries, butchers, small grocery shops
☐ Organic shops	☐ Organic shops
☐ Farm markets or street markets	☐ Farm markets or street markets
☐ Cooperatively owned or solidarity shops	☐ Cooperatively owned or solidarity shops
☐ Direct from local producer	☐ Direct from local producer
☐ Home delivery (ordered online, by phone or in-person)	☐ Home delivery (ordered online, by phone or in-person)
☐ Click this point to confirm you are not a robot	☐ Other (please specify):
☐ Other (please specify):	

MQ31

3. What are the main sources of buying your household´s OTHER FRESH PRODUCTS (MEAT, FISH, BREAD, MILK, CHEESE,ETC) before and during Covid-19?

Click as many options as are relevant!

Before Covid-19 (MQ16)	During Covid-19 (MQ17)
☐ Big supermarkets	☐ Big supermarkets
☐ Supermarkets	☐ Supermarkets
☐ Discount shops	☐ Discount shops
☐ Bakeries, butchers, small grocery shops	☐ Bakeries, butchers, small grocery shops
☐ Organic shops	☐ Organic shops
☐ Farm markets or street markets	☐ Farm markets or street markets
☐ Cooperatively owned or solidarity shops	☐ Cooperatively owned or solidarity shops
☐ Direct from local producer	☐ Direct from local producer
☐ Home delivery (ordered online, by phone or in-person)	☐ Home delivery (ordered online, by phone or in-person)
☐ Other (please specify): ____	☐ Other (please specify): ____

MQ27

4. What are the main sources of buying your household´s NON-FRESH FOOD(FROZEN, CANNEND, PRE-COOKED, DRINKS, ETC) before and during Covid-19?

Click as many options as are relevant!

Before Covid-19 (MQ18)	During Covid-19 (MQ19)
☐ Big supermarkets	☐ Big supermarkets
☐ Supermarkets	☐ Supermarkets
☐ Discount shops	☐ Discount shops
☐ Bakeries, butchers, small grocery shops	☐ Bakeries, butchers, small grocery shops
☐ Organic shops	☐ Organic shops
☐ Farm markets or street markets	☐ Farm markets or street markets
☐ Cooperatively owned or solidarity shops	☐ Cooperatively owned or solidarity shops
☐ Direct from local producer	☐ Direct from local producer
☐ Home delivery (ordered online, by phone or in-person)	☐ Home delivery (ordered online, by phone or in-person)
☐ Other (please specify): ____	☐ Other (please specify): ____

V192

5. How often does your household typically obtain the following food types before and during Covid-19?

Before Covid-19

	Less than once a fortnight or never	Between once a week and once a fortnight	Once a week	2 to 3 times a week	4 to 6 times a week	Daily
Fresh vegetables and fruit	○	○	○	○	○	○
Fresh meat and fish	○	○	○	○	○	○
Other fresh products (bread, milk, cheese, etc.)	○	○	○	○	○	○
Non-fresh food (frozen, canned, pre-cooked, drinks, etc)	○	○	○	○	○	○
Other (please specify): ____	○	○	○	○	○	○

During Covid-19 (V193)

	Less than once a fortnight or never	Between once a week and once a fortnight	Once a week	2 to 3 times a week	4 to 6 times a week	Daily
Fresh vegetables and fruit	○	○	○	○	○	○
Fresh meat and fish	○	○	○	○	○	○
Other fresh products (bread, milk, cheese, etc.)	○	○	○	○	○	○
Non-fresh food (frozen, canned, pre-cooked, drinks, etc)	○	○	○	○	○	○
Other (please specify): ____	○	○	○	○	○	○

MQ32

6. Which meals are typically prepared and eaten at home by at least one member of your household each day before and during Covid-19?

Click as many options as are relevant!

Before Covid-19 (V108)	During Covid-19 (V109)
☐ Breakfast	☐ Breakfast
☐ Midday meal	☐ Midday meal
☐ Evening meal	☐ Evening meal
☐ Random snacks	☐ Random snacks
☐ Other (please specify): ____	☐ Other (please specify): ____

Seite 12
pmeal

MQ33

7. What are the main ways your household food is prepared before and during Covid-19?

Click as many options as are relevant!

Before Covid-19 (V110)	During Covid-19 (V111)
☐ Meals from the take away shop	☐ Meals from the take away shop
☐ Take away meals from supermarket	☐ Take away meals from supermarket
☐ Ready to heat/cook meals	☐ Ready to heat/cook meals
☐ Home-made mainly from processed ingredients	☐ Home-made mainly from processed ingredients
☐ Home-made mainly from raw ingredients	☐ Home-made mainly from raw ingredients
☐ Other (please specify):	☐ Other (please specify):

Seite 13
fof

V194

8. How often do you personally eat the following food types before Covid-19?

(The During-Covid-19 options are in the next question)

Before Covid-19

	Less than once a fortnight or never	Between once a week and once a fortnight	Once a week	2 to 3 times a week	4 to 6 times a week	Daily
Fresh vegetables and fruit	○	○	○	○	○	○
Fresh meat	○	○	○	○	○	○
Fresh fish	○	○	○	○	○	○
Bread	○	○	○	○	○	○
Dairy products	○	○	○	○	○	○
Frozen food	○	○	○	○	○	○
Canned food	○	○	○	○	○	○
Ready-made meals	○	○	○	○	○	○
Cake and biscuits	○	○	○	○	○	○
Chocolate, candies	○	○	○	○	○	○
Wine, beer and other alcoholic drinks	○	○	○	○	○	○
Click “Once a week” to confirm you are not a robot	○	○	○	○	○	○
Other (please specify):	○	○	○	○	○	○

Seite 14
qc2

Seite 15
cft

V195

9. How often do you personally eat the following food types during Covid-19?

During Covid-19

	Less than once a fortnight or never	Between once a week and once a fortnight	Once a week	2 to 3 times a week	4 to 6 times a week	Daily
Fresh vegetables and fruit	○	○	○	○	○	○
Fresh meat	○	○	○	○	○	○
Fresh fish	○	○	○	○	○	○
Bread	○	○	○	○	○	○
Dairy products	○	○	○	○	○	○
Frozen food	○	○	○	○	○	○
Canned food	○	○	○	○	○	○
Ready-made meals	○	○	○	○	○	○
Cake and biscuits	○	○	○	○	○	○
Chocolate, candies	○	○	○	○	○	○
Wine, beer and other alcoholic drinks	○	○	○	○	○	○
Other (please specify):	○	○	○	○	○	○

V114

10. When eating away from home, how often do you personally use the following places before and during Covid-19?

Before Covid-19

	Less than once a fortnight or never	Between once a week and once a fortnight	Once a week	2 to 3 times a week	4 to 6 times a week	Daily
Work canteens	○	○	○	○	○	○
Restaurants, Cafés, Hotels or similar	○	○	○	○	○	○
Street vendors	○	○	○	○	○	○
Free food in hostels or similar	○	○	○	○	○	○
Other (please specify!):	○	○	○	○	○	○

V115

During Covid-19

	Less than once a fortnight or never	Between once a week and once a fortnight	Once a week	2 to 3 times a week	4 to 6 times a week	Daily
Work canteens	○	○	○	○	○	○
Restaurants, Cafés, Hotels or similar	○	○	○	○	○	○
Street vendors	○	○	○	○	○	○
Free food in hostels or similar	○	○	○	○	○	○
Other (please specify!):	○	○	○	○	○	○

V134

In the questions on the following pages, we are interested in your household's behaviour and attitudes to food during the COVID-19 pandemic in comparison with before it started.

V116

11. How much has your household´s food behaviour changed during the Covid-19 pandemic compared with before?

	Much less	A little less	No Change	A little more	Much more
How much food is eaten	○	○	○	○	○
How much money is spent on food	○	○	○	○	○
How much unpackaged food is obtained (e.g. fruit and vegetables, bread, other bulk food)	○	○	○	○	○
How much food is obtained from local producers	○	○	○	○	○
How much organic food is obtained	○	○	○	○	○
How much food is thrown away	○	○	○	○	○
How varied is the range of food eaten	○	○	○	○	○
The extent to which you plan meals and/or your grocery list in advance	○	○	○	○	○
How often new recipes and/or ingredients are used	○	○	○	○	○
The overall importance of food in your daily life	○	○	○	○	○
How far do you travel to food shops	○	○	○	○	○
How much alcohol is consumed	○	○	○	○	○

MQ34

12. Has anyone in your household missed a meal because there is not enough food in the house before and during Covid-19?

Before Covid-19 (V183)	During Covid-19 (V184)
○ Frequently	○ Frequently
○ Occasionally	○ Occasionally
○ Never	○ Never

MQ35

13. Has anyone in your household been anxious about obtaining enough food to meet their requirements before and during Covid-19?

Before Covid-19 (V185)	During Covid-19 (V186)
○ Frequently	○ Frequently
○ Occasionally	○ Occasionally
○ Never	○ Never

Seite 21
ciq

V181

14. Has your household income changed as a result of Covid-19?

- Yes
- No

V117

15. Does anyone in your household have any special dietary needs?

- Yes
- No

V118

16. Does your household stock-up on food more than in the period before Covid-19?

- Yes
- No

V119

17. Have person(s) in your household responsible for obtaining or preparing food in your household changed during the Covid-19 pandemic?

- Yes
- No

V120

18. Is any member of your household active in, or in any way closely related to, the production, processing, distribution or delivery of food to other people apart from your own household members?

- Yes
- No

V121

19. Have there been any other changes in your household's behaviour related to food before vs. during the Covid-19 pandemic?

- Yes
- No

V122

20. Have there been any other changes in your household´s attitudes to food (including any lifestyle changes) before as compared to during the Covid-19 pandemic?

- Yes
- No

Seite 22
chinc

V182
Please explain how your income has changed:

V135
Please explain which special dietary needs:

V136
What types of food do you stock more:

V137
Please explain the changes of the specific person(s) in responsibility for obtaining or preparing food:

V138
Please explain in which way member(s) of your houlsehold are related to production, processing, distribution or delivery of food:

V139
Please explain those other changes due to Covid-19:

V140
Please explain the attitude changes due to Covid-19:

Seite 23
cbehav1

V123

21. Do you expect that any of the changes in your household's behaviour and attitudes to food during Covid-19 will continue after other aspects of your household's daily life have returned to what they were before Covid-19? - Part I

Please indicate whether any of the changes in the following areas will continue:

	Definitely no	Probably no	Don´t know	Probably yes	Definitely yes	There was no change
Types of shops used	○	○	○	○	○	○
Purchasing frequency	○	○	○	○	○	○
The money spent on food	○	○	○	○	○	○
Behaviour concerning growing own food	○	○	○	○	○	○
Other means of obtaining food	○	○	○	○	○	○
Types of food and dishes	○	○	○	○	○	○
Behaviour concerning food waste	○	○	○	○	○	○
Click "Probably no" in this line to confirm you are not a robot	○	○	○	○	○	○

Seite 24
cbehav2

V191

22. Do you expect that any of the changes in your household's behaviour and attitudes to food during Covid-19 will continue after other aspects of your household's daily life have returned to what they were before Covid-19? - Part II

Please indicate whether any of the changes in the following areas will continue.

	Definitely no	Probably no	Don´t know	Probably yes	Probably yes	There was no change
Alcohol consumption	○	○	○	○	○	○
Preparing food	○	○	○	○	○	○
Where food is eaten	○	○	○	○	○	○
The person(s) in your household responsible for food	○	○	○	○	○	○
Travel distance to food shops	○	○	○	○	○	○
Obtaining food from local producers	○	○	○	○	○	○
Overall attitudes towards food	○	○	○	○	○	○
Other (please specify):	○	○	○	○	○	○

Seite 25
chreg

MQ26

23. Which changes have there been in local and national regulations, commercial or other changes resulting from the Covid-19 pandemic beyond your control?

	Yes	No	Don´t know
Travel and movement restrictions	○	○	○
Closure or restrictions on public transport	○	○	○
Closure of restaurants, cafés, canteens	○	○	○
Closure of your (physical) workplace	○	○	○
Partial or full closure of school, educational institutions, day-care institutions	○	○	○
Closure of other public places	○	○	○
Restrictions on numbers of people in one place	○	○	○
Other (please specify):	○	○	○

MQ38

If yes, please indicate the impact of these changes on your household´s behaviour and attitudes to food below.

test frage 21

Seite 26
infhouse

V126

24. Do any members of your household have Covid-19 or symptoms consistent with Covid-19 (or have they had these)?

○ Yes
○ No

V127

25. Are any members of your household in isolation or in quarantine because of Covid-19 (or have they been)?

○ Yes
○ No

V143

26. Are any members of your household in hospital because of Covid-19? (or have they been)?

○ Yes
○ No

test frage 23

Seite 27
rp

V128

27. What is your own perception of the risks of Covid-19?

	Very low	Low	Medium	High	Very high
The likelihood of any member of your household to become infected by the virus.	○	○	○	○	○
The likely severity of the virus for any member of your household (please refer to the person with the highest likely severity).	○	○	○	○	○
The level of your anxiety concerning the potential impact of the virus on your household.	○	○	○	○	○
Other (please specify):	○	○	○	○	○

Seite 28
dt10

V180

To end this survey, we would like to know some facts about you. (Note: only you as respondent)

Seite 29
soc

V129

28. Your Gender

[Please choose]

V145

29. Your age in years:

[Please choose]

V131

30. What is the highest educational qualification you have personally obtained?

[Please choose]

V132

31. How many people in the following age groups live in your household (that your previous answers about food refer to), including yourself:

0-4 Years

5-9 Years

10-19 Years

20-29 Years

30-39 Years

40-49 Years

50-59 Years

60-69 Years

70-79 Years

Over 79 Years

V130

32. Please indicate your postcode:

Seite 30
country

Seite 31

MQ37

33. Please choose your country here, if you are coming from Europe:

[please choose]

eu_country_dropdown

MQ40

34. Please choose your country here, if you are coming from outside Europe:

[please choose]

non_eu_dropdown

Seite 32
gt1

Seite 33
bl

MQ45

- Schleswig-Holstein
- Hamburg
- Niedersachsen
- Bremen
- Nordrhein-Westfalen
- Hessen
- Rheinland-Pfalz
- Baden-Württemberg
- Bayern
- Saarland
- Berlin
- Brandenburg
- Mecklenburg-Vorpommern
- Sachsen
- Sachsen-Anhalt
- Thüringen

Seite 34
quoteger

Seite 35
gt2

Seite 36
screenoutger

Bitte klicken Sie hier auf weiter!

||WEITER||

Bitte klicken Sie hier auf weiter!

||WEITER||

Letzte Seite

Thank you for completing this questionnaire!

We would like to thank you very much for helping us.

Please add your email address here if you wish to receive more information about this study:

submit

Your answers were transmitted, you may close the browser window or tab now.

Deutsches Institut für Lebensmitteltechnik e. V.
